# Highly efficient transgene‐free ErCas12a RNP‐protoplast genome editing and single‐cell regeneration in *Nicotiana benthamiana* for glyco‐engineering

**DOI:** 10.1111/pbi.70141

**Published:** 2025-06-16

**Authors:** Lukas C. Blumberg, Geartsje M. Bakker, Alex van der Kaaij, Élyse Gariépy, Henri van de Geest, Erik J. Slootweg, Ferdinand C. O. Los, Lotte B. Westerhof, Ruud H. P. Wilbers

**Affiliations:** ^1^ Laboratory of Nematology, Plant Sciences Department Wageningen University and Research Centre Wageningen The Netherlands; ^2^ Hudson River Biotechnology B.V. Wageningen The Netherlands

**Keywords:** CRISPR, ribonucleoprotein, protoplast(s), ErCas12a, *Nicotiana benthamiana*, β‐hexosaminidases

## Abstract

*Nicotiana benthamiana* serves as a unique platform for biopharmaceutical production, offering advantages such as efficient and scalable protein synthesis. In addition, custom N‐glycans can be engineered on biopharmaceutical glycoproteins. Yet, plant‐native glycosyltransferases and glycoside hydrolases need to be removed to prevent undesired modifications of tailored N‐glycans. CRISPR‐based systems offer tremendous potential; however, the ploidy of the allotetraploid *N. benthamiana* can make genome editing challenging when attempting to knock out multiple undesired enzymes using transgenes. Here, we report a highly efficient CRISPR ribonucleoprotein (RNP)‐protoplast genome editing strategy for rapid, single‐generation platform engineering. We delineate the editing characteristics of ErCas12a RNPs and apply hydrogel protoplast immobilization to characterize true single‐cell regeneration. We target three β‐hexosaminidases responsible for removing terminal GlcNAc and/or GalNAc residues from N‐glycans and verify their inactivity via MALDI‐TOF‐MS N‐glycan analysis. We achieve up to 89.6%, 95.3% and 86.5% on‐target editing in the absence of off‐target editing. We demonstrate the feasibility of low cell density (10^4^ ml^−1^) regeneration of individual CRISPR‐edited protoplasts in 12–14 weeks, carrying intended tetra‐allelic and/or deca‐allelic mutations while maintaining monoclonality. Despite the occurrence of genome duplications during the single‐cell regeneration of *N. benthamiana* protoplasts, high‐efficiency genome editing paired with shoot induction frequencies exceeding 89% facilitated the ubiquitous identification of desired β‐hexosaminidase mutants. We anticipate that this genome‐editing method will rapidly advance glyco‐engineering in polyploids such as *N. benthamiana*.

## Introduction

Plant‐production systems are increasingly recognized for their cost‐effective production of complex biopharmaceutical proteins (molecular farming) compared to microbial and mammalian systems (Ortega‐Berlanga and Pniewski, 2023; Schillberg and Finnern, 2021). Using plants as scalable ‘bioreactors’ avoids the need for advanced production facilities. Most importantly, plants can be modified to produce uniform N‐glycan compositions, resulting in consistent quality of therapeutic glycoproteins, reducing immunogenic responses and increasing these products' efficacy and safety profile (Bosch *et al*., 2013; Li *et al*., 2016; Strasser *et al*., 2008). Additionally, plants' simpler N‐glycosylation pathways and the ability to modify their glycosylation machinery make them a highly versatile platform for glycoprotein production (Schoberer and Strasser, 2018; van der Kaaij *et al*., 2022).

N‐glycosylation is the decoration of asparagine with carbohydrate structures within the consensus amino acid sequence N‐X‐S/T (where X can be any amino acid except proline). N‐glycans significantly influence eukaryotic protein characteristics, such as folding, solubility, stability, localization, half‐life and many others (Hebert *et al*., 2014). In mammals, N‐glycans also function in cell signalling and as receptor anchors, modulating processes such as antibody effector functions (Dennis *et al*., 2009). Most glycosylated biopharmaceuticals are recombinant human glycoproteins, especially antibodies (Walsh, 2018). Additionally, non‐human glycoproteins, such as immune‐modulating glycoproteins secreted by parasitic worms (helminths), have shown great potential as therapeutics against immune‐related diseases or as anti‐helminthic vaccines (Bunte *et al*., 2022). Importantly, the therapeutic effectiveness of these glycoproteins is highly dependent on their native glycan structures (Bunte *et al*., 2022; van der Zande *et al*., 2021; Zwanenburg *et al*., 2023).

In the past, plants such as *Nicotiana benthamiana* have been engineered to produce a wide range of custom N‐glycan structures; these include high‐mannose N‐glycans (Krahn *et al*., 2017; Roychowdhury *et al*., 2018), N‐glycans devoid of core α1,3‐fucose and β1,2‐xylose (Jansing *et al*., 2019; Strasser *et al*., 2008), Lewis X or GalNAc‐extended N‐glycans (Wilbers *et al*., 2017) and more (Schoberer and Strasser, 2018). Despite the advancements in engineering *N. benthamiana* for a range of N‐glycan structures, the reconstitution of an important helminth glycan motif, referred to as LacDiNAc (LDN; consisting of a GlcNAc substituted with GalNAc), faces significant challenges. Unfortunately, plant‐native glycoside hydrolases can severely hamper the yield of these glycoproteins with a defined LDN‐glycan composition. Recently, β‐hexosaminidases have been identified to trim off terminal GlcNAc and/or GalNAc from engineered N‐glycans in *N. benthamiana* (Alvisi *et al*., 2021; Shin *et al*., 2017); therefore, to improve glyco‐engineering efficiency, the β‐hexosaminidase genes must be knocked out.

A wide range of biotechnological tools is available for genome editing of *N. benthamiana* (Ali *et al*., 2015; Banakar *et al*., 2021; Hsu *et al*., 2020; Li *et al*., 2013; Nagahara *et al*., 2021; Nekrasov *et al*., 2013; Sheludko *et al*., 2007; Yin *et al*., 2015). The most widely adopted gene editing strategy involves delivering transgenes encoding the CRISPR machinery into plant cells via *Agrobacterium tumefaciens‐*mediated gene transfer (Laforest and Nadakuduti, 2022). While this method is highly variable, it has demonstrated success on many occasions (Sandhya *et al*., 2020). However, while this technique is broadly applicable, achieving GMO‐free status requires transgene removal through segregation, which requires additional breeding and screening efforts. In addition, the segregation process becomes increasingly complex when multiple copies of the transgene are present in the genome, with multiple genome‐editing targets, in polyploids, and is not feasible in clonally propagated crops. Furthermore, transgenic genome editing using multicellular explants for plant regeneration can result in mosaic plants, adding additional constraints to the segregation and selection process (Jang *et al*., 2016; Jansing *et al*., 2019; Komatsu *et al*., 2020; Pena *et al*., 1995; Song *et al*., 2022).

Exploring non‐transgenic strategies may be advantageous for the broader application of CRISPR in commercial settings, including plant molecular farming. While the final production of biopharmaceuticals must remain physically contained, other stages of the process, such as biomass bulking and material transfer, would face fewer regulatory containment constraints. Ribonucleoprotein (RNP), which is a pre‐assembled complex of endonucleases and guide RNAs, delivery to protoplasts offers a streamlined technique for precise and efficient genome editing. It obviates the need for *in vivo* synchronized expression and complexation of endonucleases and single‐guide RNAs (sgRNA) since RNPs can be produced and pre‐assembled *in vitro*, offering more precise control over the amount and timing of delivery to plant cells (Woo *et al*., 2015). This controllability lends itself to highly efficient multiplex genome editing strategies. In contrast to transgenic methods, where low temperatures required for plant growth can impede Cas enzyme activity, the transient nature and setup of the RNP‐protoplast system allow short, elevated temperature treatments to enhance editing efficiency without the loss of protoplast viability (Zhang *et al*., 2022). Moreover, the transient exposure due to the singular supply of RNPs, followed by the degradation *in vivo*, may reduce the risk of off‐target editing while alleviating transgene concerns and outcrossing needs (Modrzejewski *et al*., 2020). Lastly, monoclonal regeneration from CRISPR‐edited protoplasts overcomes genetic mosaics, enabling single‐generation transgene‐free editing (Eigel and Koop, 1989; Scintilla *et al*., 2022), positioning RNP delivery as a useful tool for rapid commercial genome editing.

Although RNP genome editing shows potential, the need for rapid and efficient editing within the short duration of protoplast RNP exposure is constrained by genetic redundancy in polyploids, which limits target site RNP accumulation (Zhang *et al*., 2021a). The absence of selection markers further compounds the necessity of high‐efficiency gene editing to minimize the cost and labour of identifying potential mutants. Furthermore, the successful regeneration of edited protoplasts into plants remains a challenge that exhibits species‐specific and genotype‐dependent constraints (Jeong *et al*., 2021; Prange *et al*., 2010). Although RNP delivery holds promise for efficient and specific CRISPR‐mediated genome editing in polyploid plants, it still requires overcoming hurdles related to editing efficiency and regeneration capacities.

This study aimed to address these issues by developing a highly efficient transgene‐free CRISPR‐ErCas12a (MAD7) editing system in *N. benthamiana* and demonstrating its utility for glyco‐engineering applications. We accomplished near‐complete delivery of RNPs targeting all (allelic) variants of three β‐hexosaminidase genes—*Nbhexo1*, *Nbhexo2* and *Nbhexo3*, which are key in N‐glycan modification (Alvisi *et al*., 2021; Shin *et al*., 2017), via PEG‐Ca^2+^ transfections of protoplast populations. Using Oxford Nanopore Technologies (ONT) sequencing of the targeted loci and predicted off‐targets, we detected high on‐target editing efficiencies of up to 89.6%, 95.3% and 86.5% without notable off‐target effects. Our results establish that the majority of ErCas12a gene editing occurs within the initial 24 h post‐transfection and that temperatures beyond 31 °C are not essential for high‐efficiency editing. Although we immobilize the transfected protoplasts in alginate at cell densities five times lower compared to other papers in the literature, we document proto‐callus shooting efficiencies exceeding 89%, facilitating the regeneration of tetra‐allelic and deca‐allelic edited, monoclonal mutants free of mosaicism within 10–14 weeks from single cell to rooted shoot. Despite the occurrence of genome duplications in 50% of single‐cell regenerated CRISPR mutants, the high efficiency of the here presented system facilitated the easy identification of the desired mutant lines. These mutants exhibited altered N‐glycan profiles, confirming a direct genotype‐to‐phenotype relationship. The established genome editing method is straightforward and manageable for individual scientists to adopt and utilize at scale. These results validate the potential of CRISPR‐ErCas12a RNP editing as a precise and potent tool for advancing the broad application of CRISPR‐based genome editing in *N. benthamiana*.

## Results

### Highly efficient internalization and nuclear localization of mNeonGreen‐tagged ErCas12a‐RNPs achieved by PEG‐based transfection within 4–24 h post‐delivery

**Figure 1:**
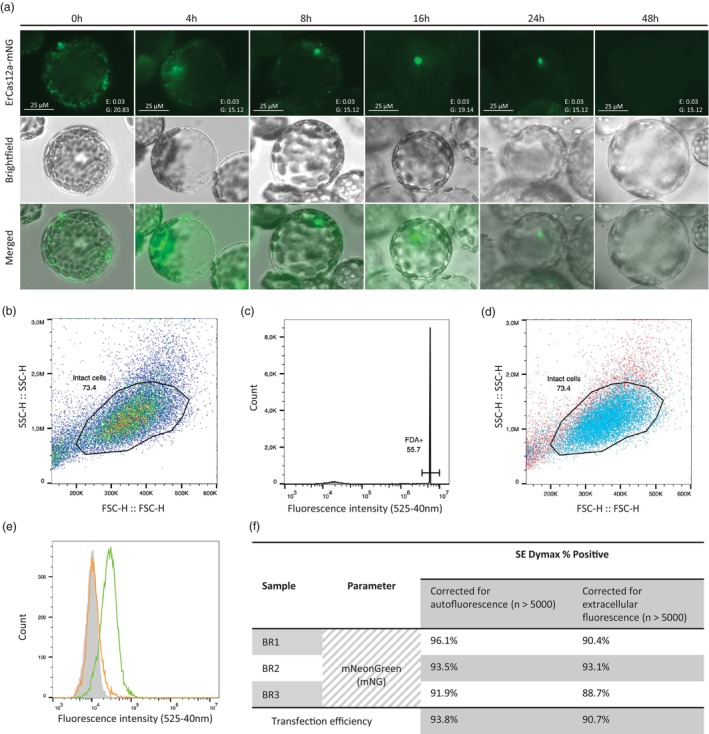
Protoplast transfection and ErCas12a‐mNG tracking. (a) Representative images of *Nicotiana benthamiana* protoplasts transfected with mNG‐tagged ErCas12a‐RNPs and imaged over the first 48 h following PEG‐mediated transfection. *E* = Exposure time, *G* = Gain, scale bar = 25 μm. (b) Scatterplot of an ungated population of mock transfected (+PEG/−ErCas12a‐mNG) *N. benthamiana* protoplasts, (*Y*‐axis) side scatter—height, (*X*‐axis) forward scatter—height, (Circle) events with the correct granularity indicating intact protoplasts. (c) Histogram of an ungated, mock transfected (+PEG/−ErCas12a) population of FDA‐stained *N. benthamiana* protoplasts (Y‐axis) absolute count of measured events, (X‐axis) fluorescence intensity (525–540 nm). (d) Scatterplot of a population of mock transfected (+PEG/−ErCas12a‐mNG), FDA‐stained *N. benthamiana* protoplasts, (Y‐axis) side scatter—height, (X‐axis) forward scatter—height, (Gate) events with the correct granularity indicating intact protoplasts, (Blue) FDA‐positive protoplasts and (Red) FDA‐negative protoplasts. (e) Flow cytometry data of a population of *N. benthamiana* protoplasts gated on granularity and size, (Grey) mock‐transfected protoplast population (+PEG/−ErCas12a‐mNG), (Orange) PEG‐less‐transfected protoplast population (−PEG/+10 μg ErCas12a‐mNG), (Green) RNP‐transfected protoplast population (+PEG/+10 μg ErCas12a‐mNG). (f) Calculation of transfection efficiencies per independent biological replicate (BR) in comparison to mock‐transfected cells (correction for autofluorescence) and transfection in the absence of PEG (correction for extracellular fluorescence), using the SE Dymax % Positive statistics (*n* > 5000).

To allow efficient editing, the transfection and subsequent nuclear localization of the RNPs need to be effective. Therefore, mNeonGreen (mNG) was C‐terminally fused to ErCas12a to assess the timing of ErCas12a‐RNP localization and protein persistence in *N. benthamiana* protoplasts. The images presented in Figure 1a are representative of these dynamics. The fluorescent tag was chosen because of its monomeric nature and excellent performance as a fusion tag (Shaner *et al*., 2013). Upon transfection, mNG‐tagged RNPs associated with the protoplast plasma membranes, followed by internalization and nuclear localization starting between 0 and 4 h post‐transfection, with clear nuclear association of the mNG‐tagged RNPs observable at the 4‐h mark. Peak nuclear association was observed between 16 and 24 h, after which the nuclear signals of mNG‐tagged RNPs reduced and were absent by 48 h, as depicted in Figure 1a. These results suggest that primary nuclear RNP activity likely occurs within the first 24 h after transfection.

To determine PEG‐mediated transfection efficiency, flow cytometry data were generated on populations of ErCas12a‐mNG transfected *N. benthamiana* protoplasts by measuring the intracellular fluorescence in 1 × 10^4^ events per sample starting at 2 h post‐transfection across three biological replicates (Figures 1b–f and S1a–l). Background autofluorescence was controlled for by using mock‐transfected protoplasts (+PEG/−ErCas12a‐mNG), and non‐internalized ErCas12a‐mNG fluorescence was addressed with protoplast samples exposed to ErCas12a‐mNG in the absence of PEG (−PEG/+ErCas12a‐mNG).

After establishing gating to selectively measure intact, living protoplasts (Figures 1b–d and S1a–i), histograms were generated to depict fluorescence intensities at 525/40 nm for the different transfection conditions in non‐FDA‐stained samples (Figures 1e and S1j–l). The marked increase in fluorescence intensity in ErCas12a‐mNG transfected samples over mock‐transfected samples indicates that most of the measured events represent protoplasts with internalized ErCas12a‐mNG (Figures 1e and S1j–l). Overlap correction between mock‐transfected and ErCas12a‐mNG transfected protoplasts was used during the transfection efficiency quantification to correct for false positives resulting from chloroplast autofluorescence (Figures 1e and S1j–l). Minor fluorescence shifts in −PEG/+ErCas12a‐mNG samples suggest minimal membrane‐associated ErCas12a‐mNG without internalization, confirmed by the slight shoulder pattern compared to mock samples (Figure 1e).

The transfection efficiency, defined as the fraction of mNG‐positive cells within the gated population, was calculated by normalizing and subtracting false positives related to chloroplast autofluorescence, as shown in Figure 1f. Using the SE Dymax % Positive statistic, an average of 93.8% of protoplasts had internalized ErCas12a‐mNG when correcting for chloroplast autofluorescence. This value slightly decreased to 90.7% using transfections in the absence of PEG. Based on the quantification of transfection efficiency, we conclude that the exposure of *N. benthamiana* protoplasts to 20% PEG for 20 min is sufficient to achieve internalization of ErCas12a‐mNG in at least 90% of the transfected protoplasts. This high efficiency is crucial for ensuring most protoplasts will undergo genome editing by the delivered RNPs. Additionally, the decrease in nuclear mNG fluorescence after 24 h points to the early post‐transfection window as essential for editing before RNP degradation sets in.

### Guide‐dependent highly efficient editing of β‐hexosaminidases in *N. benthamiana* protoplast populations

**Figure 2:**
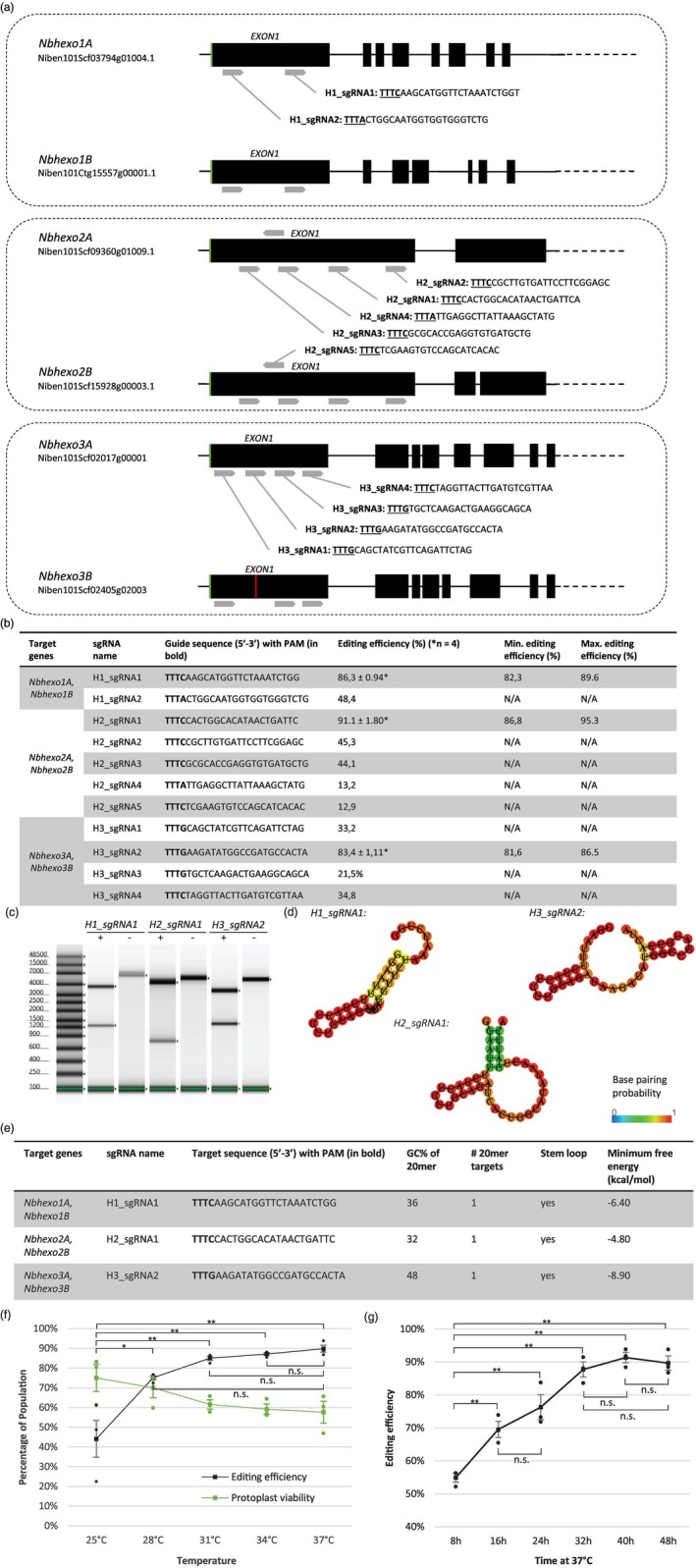
Schematic shows sgRNA location and design, characteristics, *in vitro* and *in vivo* efficiencies. (a) Gene structure of *Nbhexo1* (*Nbhexo1A, Nbhexo1B*), *Nbhexo2* (*Nbhexo2A, Nbhexo2B*) and *Nbhexo3* (*Nbhexo3A, Nbhexo3B*). (Green) Start codon on exon 1 of the open reading frame reported by Shin *et al*. (2017) and Alvisi *et al*. (2021). (Grey) Locations of the designed universal sgRNAs targeting both HEXO homoeologs in allotetraploid *Nicotiana benthamiana* with sgRNA name and sequence indicated with arrows, PAM in bold and underscored. (Red) SNP on exon 1 of *Nbhexo3B* abolishing in vivo activity of H3_sgRNA2. (b) Average, minimum and maximum transient editing efficiencies of *Nbhexo1*, *Nbhexo2* and *Nbhexo3* targeting sgRNAs in *N. benthamiana* protoplasts as determined by ONT‐NGS at 5000× coverage per target site. *Indicates ±SE of four independent biological replicates. Transient editing efficiencies in percent of edited reads over total mapped reads. (c) *In vitro* ErCas12a‐RNP cleavage assay of best‐performing sgRNAs per target gene. (d) RNAfold predicted sgRNA structures, including base‐pairing probabilities. (e) sgRNA guide sequences, target genes, GC percentage, number of 20mer targets, presence of a stem loop and minimum free energy. (f) (Black) Transient editing efficiencies of sgRNA H2_sgRNA1 in *N. benthamiana* protoplasts incubated at various temperatures for 48 h, determined by ONT‐NGS at 5000× coverage per target site on three independent biological replicates (*n* = 3). Transient editing efficiencies in percent of edited reads over total mapped reads, error bars represent SE (Green) Protoplast viability after transfection and 48 h incubation at various temperatures determined using a fluorescence cell counter and fluorescein diacetate staining on three biological replicates (*n* = 3), error bars represent SE. Editing efficiencies are compared using an ANOVA followed by Tukey HSD. Significant differences are indicated using one asterisk (*P* < 0.05) and two asterisks (*P* < 0.01). (g) Transient editing efficiencies of sgRNA H2_867_▵1 in *N. benthamiana* protoplasts at various time points during the incubation at 37 °C determined by ONT‐NGS at 5000× coverage per target site on three independent biological replicates (*n* = 3). Transient editing efficiencies in percent of edited reads over total mapped reads. Editing efficiencies are compared using an ANOVA followed by Tukey HSD. Significant differences are indicated using two asterisks (*P* < 0.01).

Literature states editing efficiencies are highly dependent on the genomic location of the target sequence (Weiss *et al*., 2022), prompting the evaluation of multiple sgRNAs per genetic target. A total of 11 sgRNAs targeting both homoeologs of either *Nbhexo1*, *Nbhexo2* or *Nbhexo3* were designed and individually screened to determine transient editing efficiencies and identify guide‐dependent variation (Figure 2a,b, Data S2). After 48 h, the RNP‐transfected protoplasts were harvested, and the target regions of interest were amplified and sequenced at 5000× coverage using ONT‐NGS. Editing efficiencies at the target sites were quantified by the percentage of ONT‐NGS reads showing indels within an 8‐nucleotide window surrounding the PAM‐distal ErCas12a cleavage site, corrected for sequencing errors (ø < 1%), using wild‐type (WT) DNA controls and the same settings.

All 11 sgRNAs demonstrated *in vivo* activity at their respective target sites, ranging from 12.9% (H2_sgRNA4) up to 95.3% (H2_sgRNA1) (Figure 2b). The most effective sgRNA for each target gene was screened in four independent biological replicates, revealing efficiencies of 86.3% ± 0.94% (*n* = 4) for H1_sgRNA1, 91.1% ± 1.80% (*n* = 4) for H2_sgRNA1 and 83.4% ± 1.11% (*n* = 4) for H3_sgRNA2 (Figure 2b). No off‐target effects were detected at predicted sites for these sgRNAs (Figure S2a,b). ONT‐NGS of the *Nbhexo3* homoeologs resulted in the identification of a novel SNP on the first exon of *Nbhexo3B* in the available *N. benthamiana* cultivar, which was not accounted for during the sgRNA design and abolished the ability of H3_sgRNA2 to target the inactive homoeolog *Nbhexo3B* (Figure 2a). Since, at that point in time, it was unclear how transient editing efficiencies in protoplast populations related to mutation efficiencies in single‐cell regenerated plants, it was decided to select the best‐performing sgRNA per target gene while neglecting the inability of H3_sgRNA2 to target *Nbhexo3B*. As such, the sgRNAs H1_sgRNA1, H2_sgRNA1 and H3_sgRNA2 were selected for *Nbhexo1*, *Nbhexo2* and *Nbhexo3*, respectively, to be used for the final mutant making. The sgRNA characteristics, such as *in vitro* activity, GC content, RNA folding scores, minimum free energy, the number of predicted 20mer targets and availability of a stem‐loop of the best‐performing sgRNAs per target gene, are illustrated in Figures 2c–e and S3a,b.

### ErCas12as' *in vivo* activity is both temperature‐ and time‐dependent

The RNP‐protoplast system's capacity for post‐transfection temperature treatments was assessed to enhance ErCas12a's *in vivo* catalytic activity. The effect of temperature on ErCas12a activity and *N. benthamiana* protoplast viability was evaluated 48 h post‐transfection across five temperature regimes using the highly efficient H2_sgRNA1 guide. Assessments were conducted in three independent biological replicates with 1 × 10^5^ protoplasts per sample (Figure 2f).

Each temperature regime led to efficient gene editing ranging from ø 44.11% ± 9% (*n* = 3) at 25 °C up to ø 89.81% ± 3% (*n* = 3) at 37 °C after 48 h of incubation (Figure 2f). Efficiencies increased with temperature but plateaued above 28 °C, showing no significant difference beyond this point (*P* > 0.05). Conversely, protoplast viability decreased with higher temperatures, from 74.99% ± 7% at 25 °C to 57.64% ± 6% at 37 °C and plateaued beyond 31 °C (Figure 2f). These results suggest a balance between ErCas12a activity and protoplast viability is achieved with post‐transfection temperatures ranging between 28 °C and 31 °C.

However, considering that the peak nuclear localization of mNG‐tagged ErCas12a occurs within the initial 24 h post‐transfection (Figure 1a), we hypothesized that shorter elevated temperature (37 °C) incubations could reduce protoplast stress without compromising editing efficiency. To test this, editing efficiency was measured at 8‐h intervals using the highly efficient H2_sgRNA1 guide. Three independent biological replicates with 1 × 10^5^ protoplasts each were conducted (Figure 2g). The findings suggest that the majority of attainable editing activity occurs within the first 24–32 h, beyond which no significant increase in editing is observed despite sustained elevated temperatures (Figure 2g).

### Transient editing efficiencies in protoplast populations match mutant creation efficiencies

Although transient editing efficiencies in protoplast populations are useful for preliminary sgRNA efficacy assessment before engaging in prolonged regeneration processes, their translation to whole plant mutant creation may be influenced by cell heterogeneity, regeneration methods, edited cell loss in tissue culture and target edit effects on cell survival. Ascertaining whether transient protoplast editing rates are indicative of overall mutant generation efficiency is crucial to inform regeneration approaches in gene editing protocols.

To assess if editing efficiencies in protoplast populations are predictive of mutant creation in regenerated plants, CRISPR‐edited single‐cell regenerated calli were genotyped by ONT‐NGS per homoeolog of *Nbhexo1* (*n* = 10), *Nbhexo2* (*n* = 10) and *Nbhexo3* (*n* = 10) and compared to the editing efficiencies of the protoplast populations from which they were regenerated (Table 1). Calli were categorized as bi‐allelic edited, mono‐allelic edited or WT. A comparison of these genotypes with the editing efficiencies of the protoplast population (Table 1) confirmed that transient editing efficiencies of protoplasts accurately represented the efficiencies of actual mutant generation. Furthermore, most of the tested calli were expected to be bi‐allelic edited, as indicated by the near‐complete editing of the target site (85%–95%), which was consistent with the data in Table 1.

**Table 1:** Comparison of transient editing efficiencies of protoplast populations to the frequencies of retrieved mutant calli

Genome target	Biologically active^†^	Transient editing efficiencies (%)	Number of calli analysed	Number of bi‐allelic	Number of monoallelic	Number of wild type
*Nbhexo1A*	Yes	87.45	10	9/10 (90%)	0/10	1/10 (10%)
*Nbhexo1B*	No	87.46		8/10 (80%)	1/10 (10%)	1/10 (10%)
*Nbhexo2A*	No	92.13	10	10/10 (100%)	0/10	0/10
*Nbhexo2B*	Yes	95.61		10/10 (100%)	0/10	0/10
*Nbhexo3A*	Yes	83.83	10	9/10 (90%)	0/10	1/10 (10%)
*Nbhexo3B*	No	N/A		N/A	N/A	N/A

Editing efficiencies and mutant calli frequencies were determined per homeolog of *Nbhexo1*, *Nbhexo2* and *Nbhexo3* in 10 protoplast‐derived calli.

^†^ As reported by Alvisi *et al*. (2021).

### Alginate immobilization facilitates the regeneration of single ErCas12a‐edited protoplasts free of mosaicism

**Figure 3:**
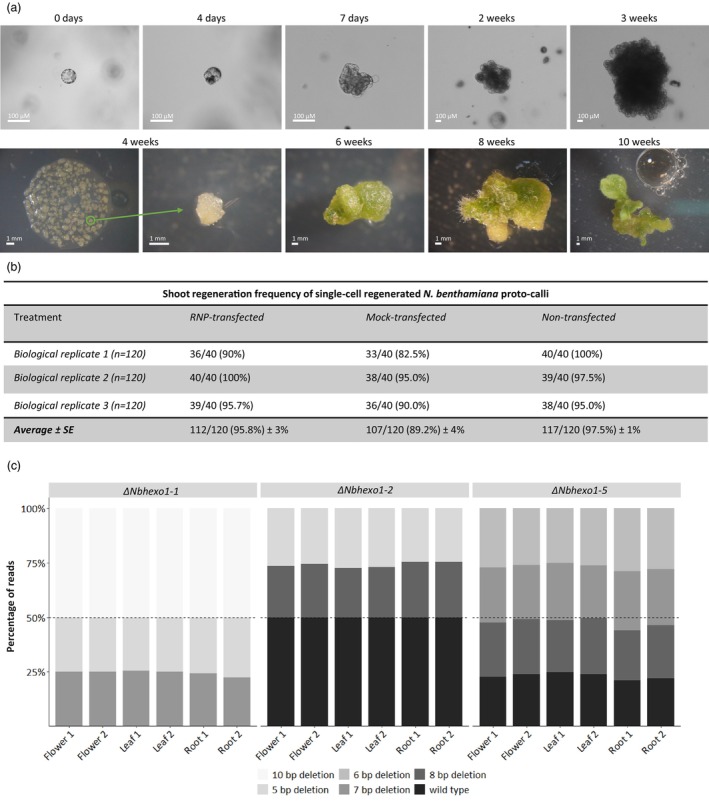
Representative pictures of low cell density protoplast regeneration and monoclonality analysis. (a) Overview of single‐cell regeneration of CRISPR‐edited *N. benthamiana* protoplasts. Starting at 0 h post‐transfection with ErCas12a and stopping 10 weeks post‐transfection. (b) Shooting efficiencies of single‐cell regenerated *N. benthamiana* protocallus across three treatments: RNP‐transfected (+ErCas12a/+PEG), Mock‐transfected (−ErCas12a/+PEG), and Non‐transfected (−ErCas12a/−PEG) averaged across three independent biological replicates (*n* = 120) ± SE. (c) Monoclonality analysis of single‐cell regenerated ErCas12a edited *Nbhexo1* mutant lines. Three *Nbhexo1* mutant lines *ΔNbhexo1‐1, ΔNbhexo1‐5*, and *ΔNbhexo1‐2* were sampled *in duplo* at three distinct locations (leaves, flowers, and roots) and genotyped by targeted ONT‐NGS. For each plant and distinct location, ONT‐NGS reads were labelled based on their editing pattern, either wild type when no editing was observed or the size of the deletion caused by the genome‐editing event.

A drawback of transgene‐mediated CRISPR is the occurrence of mosaics in regenerated mutants (Jang *et al*., 2016; Jansing *et al*., 2019; Komatsu *et al*., 2020; Li *et al*., 2016; Pena *et al*., 1995; Song *et al*., 2022). Protoplast‐based approaches mitigate this by enabling regeneration from single edited cells, thus ensuring uniformity of regenerated plants. However, regenerating individual protoplasts can be challenging due to their reluctance to re‐enter the cell cycle at low cell densities (Eigel and Koop, 1989), which is required to ensure monoclonality. Despite this, we achieved monoclonality by immobilizing RNP‐transfected protoplasts in alginate at cell densities of 10^4^ mL^−1^ and culturing them in a liquid regeneration medium supplemented with hormones and free *Nicotiana tabacum* BY‐2 cells serving as nurse cells. Although cell densities were fivefold lower compared to other protoplast regeneration protocols, the protoplasts resumed cell division within 48 h, forming micro‐calli after 1 week and growing sufficiently to be liberated from the alginate by 4 weeks before merging occurred (Figures 3a and S4d). Subsequent transfer to solid shoot induction and elongation media led to greening, expansion and differentiation, including glandular hair development within 8 weeks (Figures 3a and S4d). Shoots were isolated starting 10 weeks post‐transfection. Rooted shoots (T_0_) were obtained 12–14 weeks post‐transfection and acclimatized in the greenhouse to initiate seed production.

The frequency at which proto‐calli‐initiated shoot formation was quantified 12 weeks post‐transfection in three independent biological replicates (*n* = 3 × 120) consisting of RNP‐transfected (+ErCas12a/+PEG) (*n* = 40), mock‐transfected (−ErCas12a/+PEG) (*n* = 40) and non‐transfected (−ErCas12a/−PEG) (*n* = 40) treatments (Figure 3b). No significant difference in shoot induction frequency was observed across the three protoplast treatments. Independently of the treatment, we achieved average shoot induction frequencies exceeding 89% of the proto‐calli population.

To confirm the monoclonality and genetic consistency of mutants regenerated from single ErCas12a‐edited protoplasts, three *ΔNbhexo1* T_0_ lines were sampled *in duplo* at three distinct anatomical locations (leaves, flowers and roots) and genotyped by targeted ONT‐NGS (Figure 3c). The absence of mosaicism would have been confirmed if all samples from a respective T_0_ plant exhibited consistent indel patterns in a 50:50 ratio per homoeolog, regardless of the sampling location. During ONT‐NGS genotyping of the *Nbhexo1* homologues, reads with indels that could not be assigned to one of the main two haplotypes and that occurred in <2.5% of all reads were binned as they were consistent in quantity between all genotyping samples of a particular T_0_ plant, and therefore, it was excluded that these originate from mosaicism and instead resulted from sequencing errors.

After removing these binned reads, ONT‐NGS of the *Nbhexo1* homoeologs showed consistent indel patterns in all sampled tissues within a T_0_ line, with genotyping of *ΔNbhexo1‐1* revealing specific bi‐allelic mutations on both homoeologs, aligning with a 50:50 ratio (Figure 3c). Specifically, genotyping of *ΔNbhexo1‐1* (Figure 3c) demonstrated the presence of a −10/−10 bi‐allelic mutation on *Nbhexo1A*, as no WT allele was picked up, whereas a −7/−5 bi‐allelic mutation was found for *Nbhexo1B*. The retrieved read coverage per indel pattern followed the expected 50:50 ratio with minimal variation, which was likely caused by PCR amplification bias and/or nanopore sequencing bias. These results validated the use of our approach to produce monoclonal, transgene‐free CRISPR mutants from edited protoplasts within 10–14 weeks.

### Genome duplication events linked to tissue culture are not exacerbated by genome editing

To address concerns about potential genome duplications during protoplast‐based CRISPR gene editing, we analysed the nuclear DNA content of protoplast‐regenerated *N. benthamiana* lines. Flow cytometry was utilized to compare the nuclear‐DNA content of protoplast‐regenerated *N. benthamiana* lines relative to seed‐grown WT *N. benthamiana* plants.

**Figure 4:**
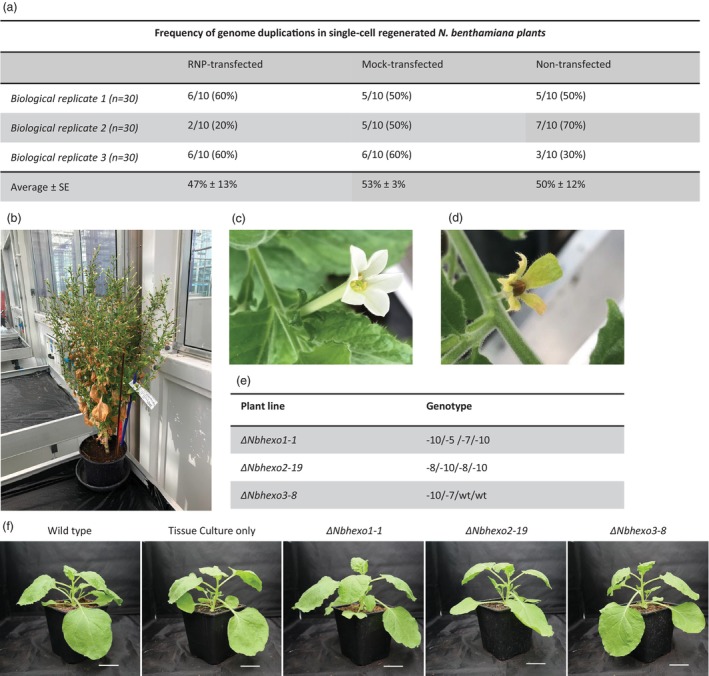
Ploidy and phenotypic analyses of *Nbhexo* mutant lines. (a) Ploidy analysis of protoplast‐regenerated *Nicotiana benthamiana* lines. (RNP‐transfected) ErCas12a edited *Nbhexo1*, *Nbhexo2* and *Nbhexo3* protoplast‐regenerated *N. benthamiana* knockout (KO) lines. (Mock‐transfected) PEG‐treated protoplast‐regenerated *N. benthamiana* lines. (Non‐transfected) Tissue culture only, regeneration of non‐transfected *N. benthamiana* protoplasts. In three independent biological replicates (*n* = 30) (b–d) T0 generation of ErCas12a edited *Nbhexo3* protoplast‐regenerated *N. benthamiana* KO lines during seed production, (b) whole plant, (c) flower, (d) seedpod. (e) Genotypes of *Nbhexo1*, *Nbhexo2* and *Nbhexo3* protoplast‐regenerated *N. benthamiana* KO lines used for recombinant glycoprotein expression and glycan structure analysis, numbers represent the nucleotide deletion size on each allele of *Nbhexo1*, *Nbhexo2* and *Nbhexo3*. (f) Phenotypes of seed‐grown wild‐type *N. benthamiana*, T1 generation of protoplast‐regenerated tissue culture only *N. benthamiana*, and T1 generation of ErCas12a edited *Nbhexo1*, *Nbhexo2* and *Nbhexo3* protoplast‐regenerated *N. benthamiana* KO lines used for recombinant protein expression. Scale bar = 5 cm.

A total of 90 T_0_ shoots were analysed across three independent biological replicates (*n* = 30) for genome duplication events (Figure 4a). Each replicate included three treatments: RNP‐transfected (+ErCas12a/+PEG) (*n* = 10), mock‐transfected (−ErCas12a/+PEG) (*n* = 10) and non‐transfected (−ErCas12a/−PEG) (*n* = 10). Normal nuclear DNA quantity of the allotetraploid *N. benthamiana* was set to 6.4 pg, as reported by Narayan (1987). We observed that, on average, 50% of protoplast‐regenerated *N. benthamiana* lines exhibited genome duplication events (Figure 4a). Genome duplications occurred independently of the protoplasts' exposure to transfection agents, such as polyethylene glycol and the editing reactants, indicating that genome duplications were unrelated to the genome editing process. Furthermore, independently of the occurrence of genome duplication events, all analysed monoclonal *N. benthamiana* lines were fertile and produced seeds (Figures 4b–d and S5a–e). In addition, the RNP‐transfected protoplast‐regenerated *N. benthamiana* lines that exhibited genome duplications (*n* = 14) were genotyped to resolve whether the genome duplication occurred before or after genome editing. If genome duplications were to be a consequence of duplicated starting material, genotyping of the regenerated lines would result in four distinct editing profiles per targeted gene copy. In case of genome duplication after genome editing, only two distinct editing profiles would be identified per targeted gene copy. Surprisingly, all RNP‐edited protoplast‐regenerated *N. benthamiana* lines carrying genome duplications exhibited only two distinct editing profiles, indicating that genome duplications were not a consequence of duplicated starting material but rather a result of the tissue culture process. Although concerning, the high efficiency of the applied system facilitated the ubiquitous identification of tetra‐allelic edited *N. benthamiana* lines with normal ploidy and phenotypic appearances. These lines were used for the subsequent agroinfiltration and N‐glycan profiling (Figure 4e,f).

### Multiplex genome editing leads to deca‐allelic *ΔNbhexo123* mutants in one generation

**Figure 5:**
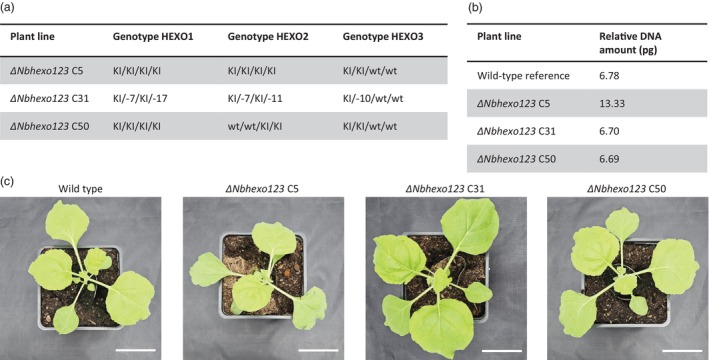
Ploidy and phenotypic analysis of three *Nbhexo123* loss‐of‐function mutant lines. (a) Genotype of three individual *ΔNbhexo123* generated (T_0_) knockout (KO) lines that were propagated and of which T_1_ lines were subsequently used for phenotypical, glycoprotein expression and glycosylation analysis. KI stands for knock‐in of the STOP‐cassette. (b) Ploidy analysis of the three regenerated *Nbhexo123* knockout lines compared to seed‐grown wild‐type (WT) *Nicotiana benthamiana*. Ploidy levels represented in nuclear DNA quantity in pg. (c) Phenotypes of seed‐grown WT *N. benthamiana* and T_1_ generation of three different ErCas12a edited *ΔNbhexo123*‐regenerated *N. benthamiana* KO lines used for recombinant protein expression. Scale bar = 5 cm.

To study the combined effects of knocking out the three different HEXO enzymes, different RNPs targeting *Nbhexo1, Nbhexo2* or *Nbhexo3* were combined in one transfection. To reduce the dependency on frameshift mutations and aid in mutant screening, a triple STOP‐cassette was designed and added during transfection simultaneously with RNPs. The cassette consisted of a primer annealing sequence, stop codons on both strands in three reading frames, and a BspTI restriction site (Figure S6a). Co‐transfection with the triple STOP‐cassette led to insertion efficiencies of up to 40% for single and triple targets, and PCR screening showed a similar insertion efficiency in regenerated shoots (Figure S6b–d). ONT‐NGS led to the identification of four mutant shoots with either the cassette or frameshift mutations in all relevant alleles, of which one shoot did not set seed and was excluded from further analysis (Figures 5a and S6e).

To assess genome duplication events, the nuclear‐DNA content of the T_0_ of the three *Nbhexo123* lines was measured and compared to seed‐grown WT *N. benthamiana plants*. While the *ΔNbhexo123* C31 and *ΔNbhexo123* C50 lines had comparable DNA content to the WT reference (6.70, 6.69 and 6.78 pg, respectively), the nuclear DNA content of the *ΔNbhexo123* C5 line was twice as high (13.33 pg) (Figure 5b). This indicated that genome duplication had occurred in the *ΔNbhexo123* C5 line only. The T_1_ plants of the three mutant lines did not seem to show any diverging phenotypes compared to the WT, except for the *ΔNbhexo123* C5, which showed slight growth deficits (Figure 5c). Nevertheless, T_1_ generations of all three lines were grown for agroinfiltration and subsequent glycan analysis.

### HEXO single and triple mutant lines are functional knockouts

To verify that each *ΔNbhexo* single and triple mutant is a true functional knockout, N‐glycan analysis was conducted using MALDI‐TOF‐MS. Alvisi *et al*. (2021) have previously uncovered the localization and substrate specificities of each NbHEXO enzyme. While NbHEXO2 and NbHEXO3 are localized in the apoplast, NbHEXO1 localizes in the vacuole. The NbHEXO enzymes also have distinct substrate specificities. NbHEXO1 and NbHEXO3 are responsible for the generation of paucimannosidic N‐glycans as they cleave terminal GlcNAc, whereas NbHEXO2 and NbHEXO3 are able to cleave GalNAc from engineered LDN‐glycans (Figure S9).

**Figure 6:**
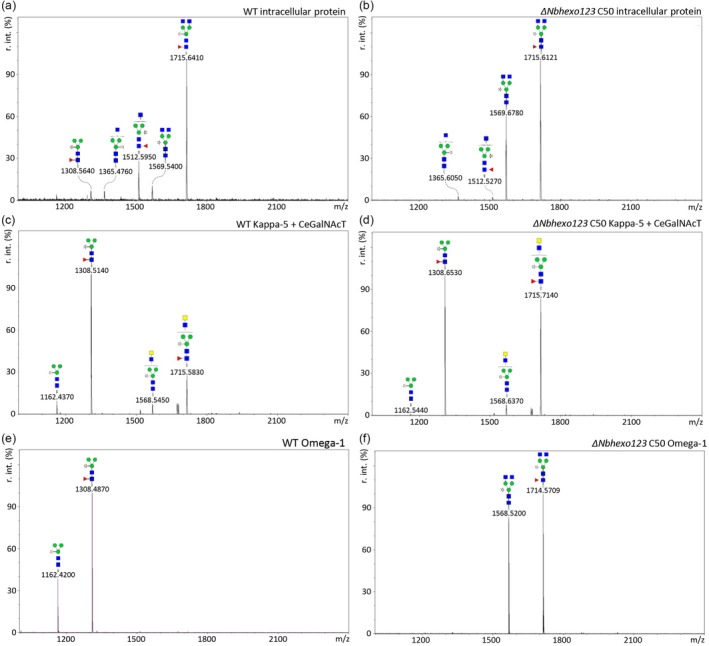
MALDI‐TOF‐MS N‐glycan analysis of protein samples from a representative *Nbhexo123* loss of function mutant line C50 and wild‐type (WT) *Nicotiana benthamiana*. Depending on the mutant line, glycans isolated from different protein samples by PNGase L digestion were analysed to verify the loss of function. Peaks of interest were labelled with the corresponding N‐glycan structure. (a, b) crude extracts from (a) WT and (b) the *ΔNbhexo123* C50 line were analysed to assess the activity of the vacuolar‐localized NbHEXO1 enzyme. (c, d) LDN engineering on Kappa‐5 with CeGalNAcT in (c) WT was compared to (d) the *ΔNbhexo123* C50 line to evaluate the activity of NbHEXO2 and NbHEXO3. (e, f) Omega‐1 was produced in (e) WT and (f) the *ΔNbhexo123* C50 line to assess the activity of NbHEXO3 and possibly NbHEXO1 in the loss of function mutant lines.

To test for NbHEXO1 enzyme activity, we isolated intracellular proteins from agroinfiltrated (single mutant) and non‐infiltrated (triple mutant) plants. The MS spectra of the crude extract from WT *N. benthamiana* (Figures 6a and S8a) show various paucimannosidic (MM), GnM and GnGn N‐glycans. In addition, various high mannose N‐glycans were observed but were not labelled, as the focus was on complex N‐glycans. By comparison, the crude extract from the *ΔNbhexo123* triple mutants (Figures 6b and S7a,b) and the *ΔNbhexo1* mutant (Figure S8b) showed a relative decline in paucimannosidic and GnM N‐glycans, whereas the GnGn structures became most dominant. This indicated that our *ΔNbhexo1* mutant lines lacked intracellular NbHEXO1 activity and that NbHEXO2 and NbHEXO3 do not affect N‐glycans on intracellular proteins. As NbHEXO1 has been shown to affect GlcNAc content on apoplastic proteins upon overexpression (Alvisi *et al*., 2021), we evaluated the N‐glycan composition of Omega‐1 upon expression in the *ΔNbhexo1* mutant line, but there was no difference compared to Omega‐1 from WT plants (Figure S7f–h).

To test for NbHEXO2 enzyme activity, we engineered LDN‐glycans on Kappa‐5 by co‐expressing CeGalNAcT. The released N‐glycans were treated with a specific β‐*N*‐acetyl‐glucosaminidase, thereby trimming terminal GlcNAc residues from the N‐glycans and only leaving structures with intact LDN. The N‐glycan composition of LDN‐engineered Kappa‐5 in WT plants (Figures 6c and S7c) was in line with previous work, revealing up to 30% LDN‐glycans (Alvisi *et al*., 2021; Wilbers *et al*., 2017). When the same LDN engineering on Kappa‐5 was performed in the *ΔNbhexo123* triple mutant lines, the number of N‐glycans carrying intact LDN motifs increased to 40–60% (Figures 6d and S8c,d), whereas in the *ΔNbhexo2* knockout, the majority of N‐glycans carried intact LDN motifs (Figure S7d). This difference can likely be attributed to differences in the efficiency of the GalNAc transfer in the different experiments as the WT control of the *ΔNbhexo2* line showed a higher LDN synthesis than the WT control of the *ΔNbhexo123* lines. Nevertheless, the results of both the single and triple mutant indicated that they lacked NbHEXO2 enzyme activity as is apparent by the increase of the LDN motif.

Finally, to test NbHEXO3 enzyme activity, we analysed the N‐glycan composition of apoplast purified Omega‐1. Omega‐1 produced in the WT background (Figures 6e and S7f) resulted predominantly in paucimannosidic N‐glycans, as reported previously (Wilbers *et al*., 2017). When Omega‐1 was produced in the *ΔNbhexo123* or *ΔNbhexo3* mutant lines (Figures 6f and S7g), the majority of the N‐glycans carried terminal GlcNAc residues, indicating that this mutant line completely lacked *ΔNbhexo3* activity in the apoplast. In addition, we analysed the efficiency of LDN‐glycan engineering in the *ΔNbhexo3* mutant line, but in contrast to the *ΔNbhexo2* mutant line, we did not observe a strong increase in LDN‐glycan synthesis (Figure S7h).

In summary, these results demonstrated the successful establishment of a method for creating monoclonal mutants within a polyploid plant species. Our system was both efficient and effective, yielding tetra‐ to deca‐allelic mutants without genome duplication events within a single generation. Phenotypical assessments corroborated that the targeted genes were successfully knocked out. Moreover, these results demonstrated that the NbHEXO enzymes do not seem to act in synergy during N‐glycosylation, as the phenotype of the *ΔNbhexo123* lines was comparable to the sum of the single *ΔNbhexo1*, *ΔNbhexo2* and *ΔNbhexo3* lines.

## Discussion

One of the core strengths of plant‐based production platforms is N‐glycan engineering. Plants' glycomes are relatively simple and homogenous, making them the perfect starting point for creating an array of customized glycan structures for enhanced biopharmaceutical functions like cellular targeting and immunogenicity (van der Kaaij *et al*., 2022). However, in proof‐of‐concept studies, several glycosidic hydrolases that can trim off engineered sugar moieties were identified (Alvisi *et al*., 2021), and therefore, we set out to knock these out of the *N. benthamiana* genome.

*Nicotiana benthamiana* is a very well known and efficient plant‐based protein production platform, and many transformation strategies exist to facilitate the required genome editing. However, given the ploidy of *N. benthamiana* and the anticipated need for multiple knockouts to generate specific glycan‐engineered mutants, traditional transgenic genome editing methods are less suitable due to inefficiencies frequently encountered in polyploids. Furthermore, transgene segregation is needed to facilitate cost‐effective biomass bulking during commercialization, prolonging the genome editing process. Consequently, we established an efficient, transgene‐free genome editing protocol that leverages single‐cell regeneration, circumventing the need for selection markers due to its high success rate and lack of mosaicism.

The developed method detailed in this paper relies on the direct delivery of the active CRISPR machinery into protoplasts. We demonstrate that ErCas12a is efficiently internalized and targeted to the nucleus, achieving near complete (up to 95%) editing within 48 h post‐transfection without off‐target editing. Our specificity assessment was tailored to the predicted off‐target sites of the employed sgRNAs rather than a broad analysis of ErCas12a specificity through systematic mismatch introduction. Using protoplast populations allowed us to capture off‐target editing frequencies that might go undetected in smaller mutant plant populations. However, the limitation to *in silico* predicted sites means potential unexpected off‐targets could be missed. Most on‐target editing variability could be attributed to genomic location and temperature incubation after protoplast transfection. Our system presents an advancement over existing (Er)Cas12a‐RNP plant editing protocols (Banakar *et al*., 2020, 2021; Dong *et al*., 2021; Kim *et al*., 2017, 2020; Sidorov *et al*., 2022; Su *et al*., 2023; Zhang *et al*., 2021b, 2023). Notably, it achieves comparable or higher genome editing efficiencies without extensive engineering of ErCas12a, even at lower temperatures and at multiple loci. Although our results unmistakably highlight the positive influence of elevated temperatures on ErCas12as *in vivo* activity, our data also reveal that post‐transfection temperatures exceeding 30 °C are not obligatory for achieving high‐efficiency genome editing (>70%) using unmodified ErCas12a in our RNP‐protoplast system. This observation starkly contrasts with transgene‐mediated (Er)Cas12a delivery to plants, which often necessitates additional (Er)Cas12a engineering to function effectively at lower temperatures (Pietralla *et al*., 2024). Consequently, we reason that RNP‐protoplast‐based systems enable a more predictable delivery and activity of the CRISPR machinery, avoiding complexities and delays associated with transgene expression systems and thereby facilitating accurate comparisons in basic research. Moreover, it opens avenues for efficient multi‐target crop improvement and trait development in polyploids, positioning ErCas12a as an appealing nuclease for RNP genome editing in a diverse array of temperature‐sensitive crops.

CRISPR genome editing in protoplasts offers the critical advantage of monoclonal regeneration, thereby eliminating the risk of genetic mosaicism (Masani *et al*., 2014; Scintilla *et al*., 2022). Despite the regeneration of CRISPR‐edited protoplasts remaining a major bottleneck for the application of transgene‐free gene editing in plants, we successfully developed an improved protocol for the regeneration of individual CRISPR‐edited *N. benthamiana* protoplasts. Our protocol employs alginate immobilization coupled with the use of *N. tabacum* nurse cells but notably diverges from conventional high‐density requirements by using a five times reduced cell density compared to existing literature (Reed and Bargmann, 2021). This advance enabled the efficient development of single cells into rooted plants within 10–14 weeks without compromising monoclonality. To our knowledge, this marks the first report of low cell density single‐cell regeneration of CRISPR‐edited *N. benthamiana* protoplasts. The applied methodology paves the way for rapid, precise and reliable applications of CRISPR technology in plant molecular farming.

Somaclonal variation, a well‐documented outcome of plant tissue culture techniques observed in protoplasts and explant regeneration (Bairu *et al*., 2011), is a significant barrier to the widespread adoption of RNP‐mediated gene editing in plant biotechnology. Influencing factors of somaclonal variation include the choice of starting material (Sharma *et al*., 2007), plant growth regulator types, concentrations and subculture frequencies (Bairu *et al*., 2006). During protoplast regeneration, variations such as single nucleotide variations (SNVs), copy number variations (CNVs), abnormal allele frequencies, loss of heterozygosity (LOH) and ploidy changes have been documented. However, incidence rates vary strongly across species and genotypes (Assani *et al*., 2006; Fossi *et al*., 2019). Genome duplications have also been reported in association with *Agrobacterium* transformations, with rates between 24% and 80% in *S. lycopersicum* (Ellul *et al*., 2003). Our study, which focused on genome duplications during protoplast regeneration due to the size of the *N. benthamiana* genome, contradicts prior findings (Lin *et al*., 2022). We show that duplications occur during tissue culture, independent of transfection agents like PEG, and are not exacerbated by its presence. Our findings suggest that in the context of *N. benthamiana* protoplast regeneration, genome duplications are not attributable to elevated ploidy levels of the starting material but rather occur after genome editing during the tissue culture process. Yet, as we developed a rapid and efficient protoplast regeneration protocol and paired it with high‐efficiency genome editing, simple accounting for duplication frequencies when scaling regeneration efforts more than compensates for this disadvantage.

Functional analysis of the *ΔNbhexo* mutant lines confirmed the non‐functionality of the NbHEXO enzymes, marking the first report of loss‐of‐function *N. benthamiana* lines for these genes. Prior work primarily involved RNAi approaches in *N. benthamiana*, T‐DNA lines in *A. thaliana* and CRISPR knockouts in rice (Liebminger *et al*., 2011; Shin *et al*., 2017, 2024). Research by Alvisi *et al*. (2021) described distinct substrate specificities for the NbHEXO enzymes. Our results show that all *ΔNbhexo* lines are functional knockouts, and each *ΔNbhexo* line provides new insights into the processing of GlcNAc and LDN on N‐glycans. Although Alvisi *et al*. (2021) reported that NbHEXO2 and NbHEXO3 can cleave GalNAc, NbHEXO3 only appears to play a minor role in LDN cleavage, possibly due to its overexpression affecting results in the analysis of Alvisi *et al*. (2021). In contrast, the knockout of NbHEXO2 significantly increased intact LDN motifs on N‐glycans. Interestingly, heightened LDN content was predominantly caused by an increase in mono‐antennary LDN and only a minor increase in di‐antennary LDN‐glycans. It was expected that NbHEXO3 caused the large fraction of monoantennary LDN within the *ΔNbhexo2* line, but the *ΔNbhexo123* did not show an increase in di‐antennary LDN‐glycans, disputing this theory. Therefore, other efforts of improving LDN synthesis on N‐glycans in plants should be explored. Previous studies showed that the additional use of a UDP‐GlcNAc C4 epimerase (to increase UDP‐GalNAc substrate availability) and UDP‐GalNAc transporters (to ensure proper Golgi localization of the substrate) did not improve LDN synthesis in the WT background (Wilbers *et al*., 2017). However, it has been shown that the C4 epimerase and transporters can improve the synthesis of GalNAc‐containing O‐glycans produced in plants (Daskalova *et al*., 2010). As LDN synthesis in the *Nbhexo123* triple mutant was still not optimal, increasing the substrate availability by adding the UDP‐GlcNAc C4 epimerase and GalNAc transporter could be a good starting point to continue research on improving the synthesis of multi‐antenna LDN motifs on N‐glycans produced in plants.

Glycan tailoring in plants often requires multi‐target genome editing, but the likelihood of obtaining the desired genotype decreases with the increasing number of targets and respective alleles. Despite the here reported high editing efficiencies for Nbhexo1 (86.3%), Nbhexo2 (91.1%) and Nbhexo3 (83.4%), the theoretical probability of retrieving a *ΔNbhexo123* deca‐allelic mutant, when excluding the probability that if one allele is edited the likelihood of a second allele being edited increases, is only 0.48%, or 0.22% when considering shooting and genome duplication frequencies. As such, we applied an NHEJ‐mediated knock‐in strategy using PCR genotyping and ONT‐NGS to identify *ΔNbhexo123* deca‐allelic mutants, circumventing the need to genotype 450 regenerants (4500 ONT‐NGS samples). Knock‐in efficiencies for a triple‐stop codon‐encoding cassette reached up to 40%. Factoring this into the earlier probability calculation does not significantly affect the probability of identifying the deca‐allelic Δ*Nbhexo123* mutant, with it dropping to 0.15% from 0.22%. However, this approach allowed PCR pre‐screening for integration events before ONT‐NGS, resulting in the identification of two deca‐allelic mutants with regular ploidy in 45 shoots (4.4%). Although these deca‐allelic mutants could no longer be considered non‐GMO or DNA‐free edited, the here performed proof of principle study facilitated analysing the processing of GlcNAc and LDN on N‐glycans in the *ΔNbhexo123* genotype.

In conclusion, we effectively engineered *N. benthamiana* for glyco‐engineering using protoplast transfection with ErCas12a‐RNPs and single‐cell regeneration, paving the way for creating plants capable of decorating biopharmaceuticals with tailored N‐glycan structures and advancing the field of plant molecular farming. From a broader perspective, our study provided an efficient transgene‐free genome editing approach for polyploid plants. This method significantly enhances efficiency, precision and speed compared to methods based on the use of transgenes. As the endonuclease and species used in the system can easily be exchanged, given a protocol for protoplast regeneration is in place, it could play a significant role in the investigation into multiplex gene editing, CRISPR‐mediated knock‐ins and precise genome editing as well as aid the adoption of CRISPR technologies in a commercial setting.

## Methods

### Plant material

*Nicotiana benthamiana* seeds were ethanol‐washed, sterilized with sodium dichloroisocyanurate (Sigma‐Aldrich, DE; 35915‐250g), and germinated on half‐strength Murashige and Skoog including vitamins medium (Duchefa, NL; M0222), supplemented with 15 g/L sucrose (Duchefa, NL; S0809.1000) and 8 g/L microagar (Duchefa, NL; M1002.1000) at pH 5.8 for 7 days at 24 °C/20 °C and 12 h light cycles. Seedlings were then grown in tissue culture pots using the same medium for 3 weeks under the same conditions.

### Genetic inventory and sgRNA design

Genomic DNA was extracted from *N. benthamiana* leaves using the DNeasy® Plant Mini Kit (Qiagen, DE; 69104), and the target regions were amplified using Phusion®High‐Fidelity Polymerase (New England Biolabs; M0530S) with appropriate primers (Table S1). Post‐PCR fragments were purified using (NucleoSpin® Gel and PCR Clean‐up kit (Macherey‐Nagel, DE; 740609.50)) and cloned into the pCR2.1 vector (Addgene; K2000‐01) using the SLiCE method (Motohashi, 2017), transformed into *Escherichia coli* TOP10 cells (ThermoFisher; C404010) and verified by colony PCR. Positive colonies were re‐amplified and Sanger‐sequenced (Macrogen, Europe). SNPs were analysed referencing the *N. benthamiana* draft genome sequence v1.0.1 (solgenomics.net) to create adjusted gene maps. These adjusted maps were used to design ErCas12a sgRNAs using Hudson River Biotechnologies' in‐house guide design software while ensuring compliance with the specifications from Inscripta (Boulder). The applied sgRNA design criteria included GC content, PAM identity, off‐targets and secondary structure.

### Recombinant ErCas12a production and in vitro complexation

A DNA template was synthesized based on the coding sequence (CDS) of ErCas12a (Inscripta) (Table S3), isolated through SgfI/PmeI digestion of pUC19‐ErCas12a and ligated into pre‐cut pFN6K‐hq using T4 ligase. His‐Tag‐TEV (Table S3) oligos were synthesized (Integrated DNA Technologies), duplexed and N‐terminally fused to AsiSI/NcoI‐digested pFN6K‐hq‐ErCas12a using T4 ligase.

The CDS of mNeonGreen (mNG) (Shaner *et al*., 2013) (Table S3) was synthesized (Integrated DNA Technologies), isolated by AsiSI/BamHI digestion of pUCIDT‐mNeonGreen and C‐terminally fused to the CDS of AsiSI/BamHI digested pFN6K‐hq‐HIS‐TEV‐ErCas12a using T4 ligase. A gBlock containing the three nuclear localization signals (SV40) (Table S3) was synthesized (Integrated DNA Technologies) and C‐terminally fused to the CDS of pFN6K‐hq‐HIS‐TEV‐ErCas12a‐mNeonGreen or pFN6K‐hq‐HIS‐TEV‐ErCas12a. *E. coli* BL21(DE3) (New England Biolabs; C2527) harbouring pFN6K‐hq‐His‐Tag‐TEV‐ErCas12a‐3xNLS or pFN6K‐hq‐His‐Tag‐TEV‐ErCas12a‐mNeonGreen‐3xNLS plasmids were inoculated in LB supplemented with 25 μg/mL Kanamycin (Duchefa, NL; K0126) and grown at 37 °C, induced in NZY Auto‐Induction LB medium (NZYtech, PT) supplemented with 25 μg/mL Kanamycin to OD_600_~6, harvested and lysed by sonication (Fisherbrand 705 Sonicator, 220‐C probe). The lysate was cleared, nucleic acids precipitated, and the supernatant was purified using affinity and size‐exclusion chromatography. ErCas12a was dialyzed, concentrated, quantified, sterilized and stored at −80 °C until use. ErCas12a RNPs were prepared by combining the endonuclease with the appropriate sgRNA (Table S2) at an equimolar ratio in 1x phosphate buffered saline (Fisher Bioreagents; 10388739). The RNP solution was incubated for 15 min at room temperature to facilitate the *in vitro* complexation of the endonuclease with the sgRNA.

### Protoplast isolation and transfection

Protoplasts from mature *in vitro* leaves were obtained by CPW (Frearson *et al*., 1973) [supplemented with 80 g/L D‐Mannitol (Duchefa, NL; M0803), 2% (w/v) Cellulase Onozuka R10 (Duchefa, NL; C8001) and 0.4% (w/v) Macerozyme Onozuka R10 (Duchefa, NL; M8002) at pH 5.8] digestion, filtered and centrifuged at 85 x g. Washed protoplasts were layered on an 18% (w/v) sucrose gradient, isolated and assessed on viability and concentration. For single‐target genome editing, 10 μg ErCas12a‐RNP was added to 1 × 10^5^ cells, transfection proceeded with 20% (w/v) PEG4000 [400 g/L PEG‐4000 (Duchefa, NL; P0804), 70 g/L D‐Mannitol (Duchefa, NL; M0803) and 0.1 M Ca(NO_3_)_2_·4 H_2_O (Carl Roth; P740.1) for 20 min], followed by treatment with 0.275 M Ca(NO_3_)_2_ * 4 H_2_O (Duchefa Biochemie, NL; 13477–34‐4). For multi‐target genome editing, three ErCas12a‐RNPs (40 μg each) targeting *Nbhexo1*, *Nbhexo2* and *Nbhexo3* with the most efficient sgRNAs of this study (Figure 2b and Table S2) were transfected into 1 × 10^6^ protoplasts. To reduce the dependency on natural frameshift induction during multi‐target genome editing, 550 pmol of a 5′ and 3′ phosphorothioate modified‐51 nt dsDNA oligo (Table S3) encoding STOP codons in all six open reading frames (ORFs) was co‐transfected with the RNPs. Post‐transfection, protoplasts were washed and cultured in K8P (Kao, 1977) supplemented with 1 mg/L NA and 0.2 mg/L BAP at 37 °C for 48 h.

### Brightfield and fluorescence protoplast imaging

Protoplasts were visualized with an EVOS M7000 system (Invitrogen; AMF7000) at 20× magnification, using 488 nm excitation and 510–560 nm detection for mNG. Exposure was 0.03 s, and gain was between 15.12 and 19.14.

### Flow cytometric transfection efficiency determination

Two hours post‐transfection, protoplasts were analysed using a CytoFLEX flow cytometer with a 488 nm laser. The protoplasts were gated on FSC‐H and SSC‐H, selecting intact protoplasts. After recording 10 000 events per sample at 30 μL/min within the gate, a mock sample was FDA‐stained for refined live cell gating in the green fluorescence channel (525–540 nm). This gating was applied to all samples to assess the shift in green fluorescence caused by mNG‐RNP internalization. The fluorescence shift in mNG‐RNP transfected versus controls was statistically analysed using the SE Dymax % Positive statistics.

### Nurse cell culture

*Nicotiana tabacum* BY‐2 cells (supplied by RIKEN BioResource Research Center, JP) were cultured in liquid full‐strength Murashige and Skoog medium (Duchefa, NL; M0221) supplemented with 30 g/L sucrose (Duchefa, NL; S0809.1000), 0.2 g/L KH_2_PO_4_ (Carl Roth, DE; T875.1), 0.1 g/L Myo‐inositol (Duchefa, NL; I0609), 1 mg/L thiamine (Duchefa, NL; T0614) and 0.2 mg/L 2,4‐D (Duchefa, NL; D0911). The packed cell volume (PCV) was determined twice a week, and a fraction of the cell suspension was transferred to fresh culture medium to achieve a desired PCV value of 2%–4%.

### Protoplast regeneration

RNP‐transfected protoplasts were centrifuged and resuspended in 2% (w/v) alginate [72.88 g/L D‐Mannitol (Duchefa, NL; M0803) and 20 g/L Alginic acid sodium salt (Sigma‐Aldrich; A0682)] to achieve a final cell density of 10^4^/mL. Thereafter, 200 μL fractions of the alginate‐protoplast suspension were cast onto CA agar [72.5 g/L D‐Mannitol (Duchefa, NL; M0803), 7.35 g/L CaCl_2_·2H_2_O (Carl Roth; HN04.1) and 8 g/L Microagar (Duchefa, NL; M1002.1000)] and left to polymerize for 30 min. Solidified disks were combined with free nurse cells at 25% v/v in 8M, and K8P supplemented with 1 mg/L NAA (Duchefa Biochemie, NL; N0903) and 0.2 mg/L BAP (Duchefa Biochemie, NL; B0904) and incubated at 37 °C in the darkness for 48 h, followed by 28 °C in the darkness for 14 days. After 14 days at 28 °C, the alginate disks were transferred to K8P without nurse cells or auxin and grown under light at 24 °C/20 °C (16/8 h photoperiod) for an additional 14 days. After 14 days under the light, calli were isolated and cultured on a solid full‐strength Murashige and Skoog medium including vitamins (Duchefa, NL; M0222), supplemented with 30 g/L sucrose (Duchefa, NL; S0809.1000), 8 g/L microagar (Duchefa, NL; M1002.1000) and 1 mg/L TDZ (Duchefa, NL; T0916) at pH 5.8, under light at 24 °C/20 °C (16/8 h photoperiod). The culture medium was refreshed every 14 days until shoot primordia began to develop, at which point the calli were transferred to a solid full‐strength Murashige and Skoog medium including vitamins (Duchefa, NL; M0222), supplemented with 30 g/L sucrose (Duchefa, NL; S0809.1000), 8 g/L microagar (Duchefa, NL; M1002.1000) and 1 mg/L 2‐iP (Duchefa, NL; D0906) at pH 5.8. Once the shoots were elongated, the calli were transferred to solid full‐strength Murashige and Skoog medium, including vitamins (Duchefa, NL; M0222), supplemented with 30 g/L sucrose (Duchefa, NL; S0809.1000) and 8 g/L microagar (Duchefa, NL; M1002.1000) free of plant growth regulators. After one subculture, shoots were isolated and then transferred to a solid full‐strength Murashige and Skoog medium including vitamins (Duchefa, NL; M0222), supplemented with 30 g/L sucrose (Duchefa, NL; S0809.1000), 8 g/L microagar (Duchefa, NL; M1002.1000) and 0.1 mg/L IBA (Duchefa, NL; I0902) at pH 5.8. Rooted shoots were deflasked and transferred to a climate‐controlled greenhouse where the plants were grown at 22 °C/21 °C for seed production.

### Transient editing efficiency determination and mutant genotyping

RNP‐transfected protoplast and CRISPR‐mutant plant DNA were extracted [NucleoSpin Plant II kit (Macherey‐Nagel, DE; 740770)] and amplified using Phusion High‐Fidelity Polymerase (New England Biolabs; M0530S), with appropriate primers (Table S1). PCR products were purified (ProNex size‐selective purification system (Promega; NG2001)) and quantified [Quant‐iT PicoGreen dsDNA kit (Invitrogen; P7589)]. Amplicons were end‐prepped [NEBNext Ultra™ II End Repair/dA‐Tailing Module (New England Biolabs; E7546)], barcoded [Native Barcoding Kit 96 V14 kit (Oxford Nanopore Technologies, UK; SQK‐NBD114.96)] and the NEB Blunt/TA Ligase Master Mix (New England Biolabs; M0367)), adapter‐ligated, and pooled. The library was loaded onto an R10.4.1 flow cell (Oxford Nanopore Technologies, UK; FLO‐MIN114) and run overnight until each sample had a minimum of 5000 reads. The amplicons were basecalled using Minknow (Oxford Nanopore Technologies, UK; v24.02.16). Reads were mapped against their reference using minimap2 (v2.27‐r1193) (Li, 2018), settings ‘‐t4 ‐‐MD ‐ay ‐xsplice ‐un ‐G 5K ‐‐cs=short’ and organized with samtools (v1.1) (Li *et al*., 2009). Variant calling was performed with freebayes (v1.3.6) (Garrison and Marth, 2012), whereby >2 nt difference to the reference sequence in the 8 nt's covering the cleavage site of ErCas12a is considered edited. The determined editing efficiencies were adjusted for sequencing errors by subtracting indel occurrence rates at the target site as determined on WT DNA, using the same settings.

### Phenotyping and ploidy analysis

T_1_ CRISPR line seeds (*n* = 7) were germinated, soil‐transferred and grown in a controlled greenhouse at 22 °C/21 °C for phenotyping. For ploidy analysis, single‐cell regenerated T_0_ *in vitro* plants were leaf‐sampled for cellular DNA content analysis by flow cytometry (Iribov B.V., The Netherlands).

### Transient protein expression, purification and N‐glycan analysis

Intracellular proteins were extracted from T1 plants of newly generated loss‐of‐function mutants or WT plants. The glycoproteins Kappa‐5 and Omega‐1 from the human parasite *Schistosoma mansoni* were transiently produced in these same plants as previously described (Wilbers *et al*., 2017). To engineer LDN‐glycans on Kappa‐5, we co‐expressed a β1,4‐*N*‐acetyl‐galactosaminyltransferase from *Caenorhabditis elegans* (CeGalNAcT) (Wilbers *et al*., 2017). Recombinant glycoproteins were expressed, purified and analysed for N‐glycan composition as previously described (Wilbers *et al*., 2017). Purified glycoproteins were dialyzed to PBS (Fisher Bioreagents; 10388739), and afterwards, protein concentrations were measured using a Pierce™ BCA protein assay kit (Thermo Fisher Scientific; 23225). For MALDI‐TOF‐MS N‐glycan analysis, 40 μg of purified Kappa‐5 or Omega‐1 or 5 μL of intracellular proteins were processed for PNGase A or PNGase L (Ludger, UK; LZ‐PNGaseL‐50‐KIT) N‐glycan release. To confirm the presence of terminal GalNAc on the N‐glycan antennae of LDN‐engineered samples, the labelled N‐glycans were treated with β‐*N*‐acetyl‐glucosaminidase S (New England Biolabs; P0744). After the enzyme treatment, samples were cleaned using a C18 ZipTip™ (MerckMillipore; ZTC18S096) and analysed using MALDI‐TOF‐MS.

## Author contributions

LCB, GMB and AK designed the experiments and were advised by ES, LBW and RHPW. LCB, GMB, AK and EG performed the experiments. LCB, GMB, AK, EG, HG, ES, LBW and RHPW analysed the data. LBW and RHPW conceived the project. LCB, GMB and AK wrote the draft manuscript that was edited by HG, ES, LBW and RHPW. FCOL and RHPW provided funding. All authors read and approved the final article.

## Funding statement

The authors declare that this study received funding from Hudson River Biotechnology B.V. In addition, this project was co‐funded by the Netherlands Organization for Scientific Research under the Netherlands organisation for scientific research Grant 16740, and by the European Union under the HORIZON EUROPE project WORMVACS2.0 (Grant Agreement No. 101080784). Views and opinions expressed are however those of the author(s) only and do not necessarily reflect those of the European Union or European Health and Digital Executive Agency (HADEA). Neither the European Union nor the granting authority (HADEA) can be held responsible for them.

## Conflict of interest

LBW, HG and EJS are employed by Hudson River Biotechnology B.V. FCOL is a shareholder of the company. The research performed in this study forms part of the Ph.D. of LCB, funded by Hudson River Biotechnology B.V., which is the developer of BioMaas, a DNA‐free CRISPR workflow. The remaining authors declare that the research was conducted without any commercial or financial relationships that could be construed as a potential conflict of interest.

## Supporting information


**Table S1** Primer sequences used in this study.
**Table S2** sgRNA sequences used in this study.
**Table S3** Synthesized sequences used in this study.

**Figure S1** Flow cytometric analysis of ErCas12a internalization.
**Figure S2** Off‐target editing evaluation at three most likely targets of H1_sgRNA1, H2_sgRNA1, and H3_sgRNA2.
**Figure S3** *In vitro* cleavage assays of ErCas12a.
**Figure S4** Low cell density single‐cell regeneration of ErCas12a edited *N. benthamiana* protoplasts.
**Figure S5** Phenotypic analysis of the generated *Nbhexo* loss‐of‐function mutants.
**Figure S6** Multiplex genome editing in *N. benthamiana* protoplasts.
**Figure S7** MALDI‐TOF‐MS N‐glycan analysis of protein samples from the generated *Nbhexo* loss of function mutants and WT *N. benthamiana*.
**Figure S8** MALDI‐TOF‐MS N‐glycan analysis of protein samples from two *Nbhexo123* loss of function mutant lines.
**Figure S9** Beta‐hexosaminidase (HEXO) activity along the secretory pathway in plants.

## Data Availability

The original contributions presented in the study are included in the article/Supplementary Materials. Further inquiries can be directed to the corresponding author.
