# Supplementary material for: Highly efficient transgene‐free ErCas12a RNP‐protoplast genome editing and single‐cell regeneration in Nicotiana benthamiana for glyco‐engineering
Source: Plant Biotechnol J. 2025 Jun 16;24(1):239–55. doi: 10.1111/pbi.70141 (PMC12854893; doi:10.1111/pbi.70141)

**Supplemental data**

**Table S1. Primer sequences used in this study.**

| **Primer Name** | **Primer sequence (5’ to 3’)** | **Primer Info** |
| --- | --- | --- |
| SL_HEXO1.1_FW | gggcgaattctgcagatAGGTCCAAAACGGGCTCCTAGT | Genetic inventory Nbhexo1, SLICE overhang lowercase |
| SL_HEXO1.1_RV | ccgccagtgtgatggatCCCTTGCCCCAAGCGATGTCAC | Genetic inventory Nbhexo1, SLICE overhang lowercase |
| SL_HEXO1.2_FW | gggcgaattctgcagatTGAAGTTTTGTGAGTGATTTGAGTGA | Genetic inventory Nbhexo1, SLICE overhang lowercase |
| SL_HEXO1.2_RV | ccgccagtgtgatggatCCTATGAGAGCTTGTGGGCAGA | Genetic inventory Nbhexo1, SLICE overhang lowercase |
| SL_HEXO2.1_FW | gggcgaattctgcagatAGTCCAGAAGATGACTCCAAGC | Genetic inventory Nbhexo2, SLICE overhang lowercase |
| SL_HEXO2.1_RV | ccgccagtgtgatggatTGATCCCCATGTTCTGAAGG | Genetic inventory Nbhexo2, SLICE overhang lowercase |
| SL_HEXO2.2_FW | gggcgaattctgcagatAATAAATACTCCTCCCCACTCTTTG | Genetic inventory Nbhexo2, SLICE overhang lowercase |
| SL_HEXO2.2_RV | ccgccagtgtgatggatTCTTGTTCCTTAACGGGGTTAC | Genetic inventory Nbhexo2, SLICE overhang lowercase |
| SL_HEXO3.1_FW | gggcgaattctgcagatGCACTCAGATTCAAATGTCTTCC | Genetic inventory Nbhexo3, SLICE overhang lowercase |
| SL_HEXO3.1_RV | ccgccagtgtgatggatAAGTGAAGATTCTCCGGTGTG | Genetic inventory Nbhexo3, SLICE overhang lowercase |
| SL_HEXO3.2_FW | gggcgaattctgcagatTTGTTCATTTTGTTTCTTGTTGC | Genetic inventory Nbhexo3, SLICE overhang lowercase |
| SL_HEXO3.2_RV | ccgccagtgtgatggatTGCATTTGAATGGACAAAATATC | Genetic inventory Nbhexo3, SLICE overhang lowercase |
| M13_FW | CTGGCCGTCGTTTTAC | Colony PCR FW primer for OneTaq |
| M13_RV | CAGGAAACAGCTATGAC | Colony PCR RV primer for OneTaq |
| HEXO1A_FW | CCAAAACGGGCTCCTAGTTAAA | Nbhexo1A specific genotyping primer |
| HEXO1A_RV | CGGGTAAAAATGGTAACTAACATGC | Nbhexo1A specific genotyping primer |
| HEXO1B_FW | CTGTCGTCAAAGGAGAGAAGCA | Nbhexo1B specific genotyping primer |
| HEXO1B_RV | GCCATGATACGCATTAACAGTAGC | Nbhexo1B specific genotyping primer |
| HEXO2A_FW | GGCTTACTTCATTTCATTTCTCTCC | Nbhexo2A specific genotyping primer |
| HEXO2A_RV | CCGTTTGGTGTAATAAGCATCC | Nbhexo2A specific genotyping primer |
| HEXO2B_FW | TTCCTTGTCTCCTTGTTATTCTTTATCTTCATTTCTCAG | Nbhexo2B specific genotyping primer |
| HEXO2B_RV | TCTTGTTCCTTTGCGGGGTTCC | Nbhexo2B specific genotyping primer |
| HEXO3A_FW | GCACTCAGATTCAAATGTCTTCC | Nbhexo3A specific genotyping primer |
| HEXO3A_RV | TTCACACCGGAGAATCTTCAC | Nbhexo3A specific genotyping primer |
| HEXO3B_FW | CGAGAGGTTGTGAGTTCGAGTC | Nbhexo3B specific genotyping primer |
| HEXO3B_RV | GAAAGCCAAATCATCTAGTTGCAG | Nbhexo3B specific genotyping primer |
| HEXO1_OFFTR_1_FW | TATACCGTCAAGGCATGCAA | Nbhexo1 predicted off-target 1 |
| HEXO1_OFFTR_1_RV | GAATGCGAGGAATGCAAACT | Nbhexo1 predicted off-target 1 |
| HEXO1_OFFTR_2_FW | TGTGGGTTTCATTTCAGCAA | Nbhexo1 predicted off-target 2 |
| HEXO1_OFFTR_2_RV | AAAAGGCCAGGTACCAGTGA | Nbhexo1 predicted off-target 2 |
| HEXO1_OFFTR_3_FW | CCAGACTAGCCCAAACCGTA | Nbhexo1 predicted off-target 3 |
| HEXO1_OFFTR_3_RV | GAAGCAATTGGGGTGAAGAA | Nbhexo1 predicted off-target 3 |
| HEXO2_OFFTR_1_FW | GTGGCAAAAATGTGTGTCGT | Nbhexo2 predicted off-target 1 |
| HEXO2_OFFTR_1_RV | CAAACACCCCATGTTTTCTG | Nbhexo2 predicted off-target 1 |
| HEXO2_OFFTR_2_FW | CGGACACTTTGACCTGTGTC | Nbhexo2 predicted off-target 2 |
| HEXO2_OFFTR_2_RV | GCTAAACCAGACCCCATTTT | Nbhexo2 predicted off-target 2 |
| HEXO2_OFFTR_3_FW | CCTTTTCCAAACAGCAAGC | Nbhexo2 predicted off-target 3 |
| HEXO2_OFFTR_3_RV | CTGTGGAAGCAAATGAAAGC | Nbhexo2 predicted off-target 3 |
| HEXO3_OFFTR_1_FW | CCTCCCATCGAGAAATATCA | Nbhexo3 predicted off-target 1 |
| HEXO3_OFFTR_1_RV | GGTTACATGGCGGTGTTCT | Nbhexo3 predicted off-target 1 |
| HEXO3_OFFTR_2_FW | CGATGAGGTACCACTGAACC | Nbhexo3 predicted off-target 2 |
| HEXO3_OFFTR_2_RV | CTCTTTTCTTTCGCAGATGG | Nbhexo3 predicted off-target 2 |
| HEXO3_OFFTR_3_FW | AGTCCGGATGCTTTACCTCT | Nbhexo3 predicted off-target 3 |
| HEXO3_OFFTR_3_RV | GTTACCCTCTCAGCAGCAAA | Nbhexo3 predicted off-target 3 |

**Table S2. sgRNA sequences used in this study.**

| **sgRNA name** | **sgRNA sequence (5’ to 3’)** | **PAM** | **sgRNA info** |
| --- | --- | --- | --- |
| H1_sgRNA1 | ggaatttctactcttgtagatAAGCATGGTTCTAAATCTGG | TTTC | Nbhexo1 targeting, backbone lowercase |
| H1_sgRNA2 | ggaatttctactcttgtagatCTGGCAATGGTGGTGGGTCTG | TTTA | Nbhexo1 targeting, backbone lowercase |
| H2_sgRNA1 | ggaatttctactcttgtagatCACTGGCACATAACTGATTC | TTTC | Nbhexo2 targeting, backbone lowercase |
| H2_sgRNA2 | ggaatttctactcttgtagatCGCTTGTGATTCCTTCGGAGC | TTTC | Nbhexo2 targeting, backbone lowercase |
| H2_sgRNA3 | ggaatttctactcttgtagatGCGCACCGAGGTGTGATGCTG | TTTC | Nbhexo2 targeting, backbone lowercase |
| H2_sgRNA4 | ggaatttctactcttgtagatTTGAGGCTTATTAAAGCTATG | TTTA | Nbhexo2 targeting, backbone lowercase |
| H2_sgRNA5 | ggaatttctactcttgtagatTCGAAGTGTCCAGCATCACAC | TTTC | Nbhexo2 targeting, backbone lowercase |
| H3_sgRNA1 | ggaatttctactcttgtagatCAGCTATCGTTCAGATTCTAG | TTTG | Nbhexo3 targeting, backbone lowercase |
| H3_sgRNA2 | ggaatttctactcttgtagatAAGATATGGCCGATGCCACTA | TTTG | Nbhexo3 targeting, backbone lowercase |
| H3_sgRNA3 | ggaatttctactcttgtagatTGCTCAAGACTGAAGGCAGCA | TTTG | Nbhexo3 targeting, backbone lowercase |
| H3_sgRNA4 | ggaatttctactcttgtagatTAGGTTACTTGATGTCGTTAA | TTTC | Nbhexo3 targeting, backbone lowercase |

**Table S3. Synthesized sequences used in this study**

| **Sequence name** | **Sequence (5’-3’)** |
| --- | --- |
| ErCas12a (CDS) | aacaacggcacaaataattttcagaacttcatcgggatctcaagtttgcagaaaacgctgcgcaatgctctgatccccacggaaaccacgcaacagttcatcgtcaagaacggaataattaaagaagatgagttacgtggcgagaaccgccagattctgaaagatatcatggatgactactaccgcggattcatctctgagactctgagttctattgatgacatagattggactagcctgttcgaaaaaatggaaattcagctgaaaaatggtgataataaagataccttaattaaggaacagacagagtatcggaaagcaatccataaaaaatttgcgaacgacgatcggtttaagaacatgtttagcgccaaactgattagtgacatattacctgaatttgtcatccacaacaataattattcggcatcagagaaagaggaaaaaacccaggtgataaaattgttttcgcgctttgcgactagctttaaagattacttcaagaaccgtgcaaattgcttttcagcggacgatatttcatcaagcagctgccatcgcatcgtcaacgacaatgcagagatattcttttcaaatgcgctggtctaccgccggatcgtaaaatcgctgagcaatgacgatatcaacaaaatttcgggcgatatgaaagattcattaaaagaaatgagtctggaagaaatatattcttacgagaagtatggggaatttattacccaggaaggcattagcttctataatgatatctgtgggaaagtgaattcttttatgaacctgtattgtcagaaaaataaagaaaacaaaaatttatacaaacttcagaaacttcacaaacagattctatgcattgcggacactagctatgaggtcccgtataaatttgaaagtgacgaggaagtgtaccaatcagttaacggcttccttgataacattagcagcaaacatatagtcgaaagattacgcaaaatcggcgataactataacggctacaacctggataaaatttatatcgtgtccaaattttacgagagcgttagccaaaaaacctaccgcgactgggaaacaattaataccgccctcgaaattcattacaataatatcttgccgggtaacggtaaaagtaaagccgacaaagtaaaaaaagcggttaagaatgatttacagaaatccatcaccgaaataaatgagctagtgtcaaactataagctgtgcagtgacgacaacatcaaagcggagacttatatacatgagattagccatatcttgaataactttgaagcacaggaattgaaatacaatccggaaattcacctagttgaatccgagctcaaagcgagtgagcttaaaaacgtgctggacgtgatcatgaatgcgtttcattggtgttcggtttttatgactgaggaacttgttgataaagacaacaatttttatgcggaactggaggagatttacgatgaaatttatccagtaattagtctgtacaacctggttcgtaactacgttacccagaaaccgtacagcacgaaaaagattaaattgaactttggaataccgacgttagcagacggttggtcaaagtccaaagagtattctaataacgctatcatactgatgcgcgacaatctgtattatctgggcatctttaatgcgaagaataaaccggacaagaagattatcgagggtaatacgtcagaaaataagggtgactacaaaaagatgatttataatttgctcccgggtcccaacaaaatgatcccgaaagttttcttgagcagcaagacgggggtggaaacgtataaaccgagcgcctatatcctagaggggtataaacagaataaacatatcaagtcttcaaaagactttgatatcactttctgtcatgatctgatcgactacttcaaaaactgtattgcaattcatcccgagtggaaaaacttcggttttgattttagcgacaccagtacttatgaagacatttccgggttttatcgtgaggtagagttacaaggttacaagattgattggacatacattagcgaaaaagacattgatctgctgcaggaaaaaggtcaactgtatctgttccagatatataacaaagatttttcgaaaaaatcaaccgggaatgacaaccttcacaccatgtacctgaaaaatcttttctcagaagaaaatcttaaggatatcgtcctgaaacttaacggcgaagcggaaatcttcttcaggaagagcagcataaagaacccaatcattcataaaaaaggctcgattttagtcaaccgtacctacgaagcagaagaaaaagaccagtttggcaacattcaaattgtgcgtaaaaatattccggaaaacatttatcaggagctgtacaaatacttcaacgataaaagcgacaaagagctgtctgatgaagcagccaaactgaagaatgtagtgggacaccacgaggcagcgacgaatatagtcaaggactatcgctacacgtatgataaatacttccttcatatgcctattacgatcaatttcaaagccaataaaacgggttttattaatgataggatcttacagtatatcgctaaagaaaaagacttacatgtgatcggcattgatcggggcgagcgtaacctgatctacgtgtccgtgattgatacttgtggtaatatagttgaacagaaaagctttaacattgtaaacggctacgactatcagataaaactgaaacaacaggagggcgctagacagattgcgcggaaagaatggaaagaaattggtaaaattaaagagatcaaagagggctacctgagcttagtaatccacgagatctctaaaatggtaatcaaatacaatgcaattatagcgatggaggatttgtcttatggttttaaaaaagggcgctttaaggtcgaacggcaagtttaccagaaatttgaaaccatgctcatcaataaactcaactatctggtatttaaagatatttcgattaccgagaatggcggtctcctgaaaggttatcagctgacatacattcctgataaacttaaaaacgtgggtcatcagtgcggctgcattttttatgtgcctgctgcatacacgagcaaaattgatccgaccaccggctttgtgaatatctttaaatttaaagacctgacagtggacgcaaaacgtgaattcattaaaaaatttgactcaattcgttatgacagtgaaaaaaatctgttctgctttacatttgactacaataactttattacgcaaaacacggtcatgagcaaatcatcgtggagtgtgtatacatacggcgtgcgcatcaaacgtcgctttgtgaacggccgcttctcaaacgaaagtgataccattgacataaccaaagatatggagaaaacgttggaaatgacggacattaactggcgcgatggccacgatcttcgtcaagacattatagattatgaaattgttcagcacatattcgaaattttccgtttaacagtgcaaatgcgtaactccttgtctgaactggaggaccgtgattacgatcgtctcatttcacctgtactgaacgaaaataacattttttatgacagcgcgaaagcgggggatgcacttcctaaggatgccgatgcaaatggtgcgtattgtattgcattaaaagggttatatgaaattaaacaaattaccgaaaattggaaagaagatggtaaattttcgcgcgataaactcaaaatcagcaataaagattggttcgactttatccagaataagcgctatctc |
| His-Tag-TEV oligo 1 | atcatcatcatcatgagaacctgtacttccaaagtagc |
| His-Tag-TEV oligo 2 | gctactttggaagtacaggttctcatgatgatgatgat |
| mNeonGreen (CDS) | atggtttctaagggtgaggaggacaacatggcttctctccctgctactcacgagctccacatcttcggttctatcaacggtgttgacttcgacatggttggtcagggtactggtaaccctaacgacggttacgaggagctcaacctcaagtctactaagggtgacctccagttctctccttggattctcgttcctcacatcggttacggtttccaccagtacctcccttaccctgacggtatgtctcctttccaggctgctatggttgacggttctggttaccaggttcaccgtactatgcagttcgaggacggtgcttctctcactgttaactaccgttacacttacgagggttctcacatcaagggtgaggctcaggttaagggtactggtttccctgctgacggtcctgttatgactaactctctcactgctgctgactggtgccgttctaagaagacttaccctaacgacaagactatcatctctactttcaagtggtcttacactactggtaacggtaagcgttaccgttctactgctcgtactacttacactttcgctaagcctatggctgctaactacctcaagaaccagcctatgtacgttttccgtaagactgagctcaagcactctaagactgagctcaacttcaaggagtggcagaaggctttcactgacgttatgggtatggacgagctctacaag |
| 3xNLS (SV40) | cccaagaagaagaggaaggtggaagatcccaagaaaaaacgcaaggtggaagatcctaagaaaaagcggaaagtg |
| Stop-oligo A | T*A*GATAGATAGCTAGACCTTACTTGACCGGATACCTATCTATCTACTTA*A*G |
| Stop-oligo B | C*T*TAAGTAGATAGATAGGTATCCGGTCAAGTAAGGTCTAGCTATCTATC*T*A |

**Supplemental Figure 5. Phenotypic analysis of the generated *Nbhexo* loss-of-function mutants. (**a) Wild-type *N. benthamiana.* (b) Tissue culture only (-PEG/-ErCas12a). (c) *ΔNbhexo1* plants. (d) *ΔNbhexo2* plants. (e) *ΔNbhexo3* plants. (n=3), scale bar = 5 cm.


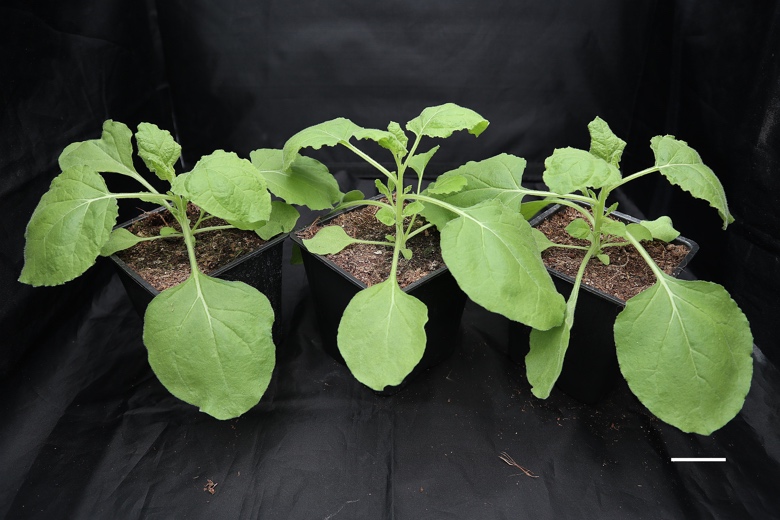


Wild type


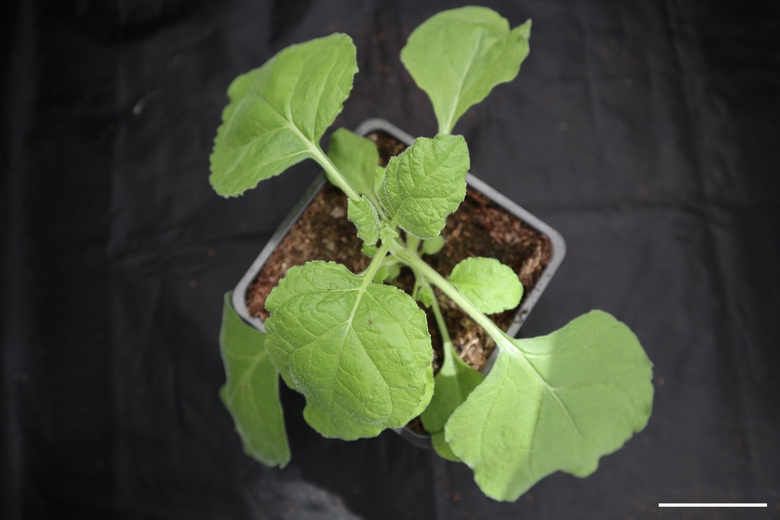

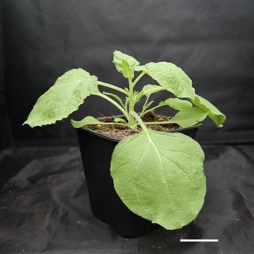

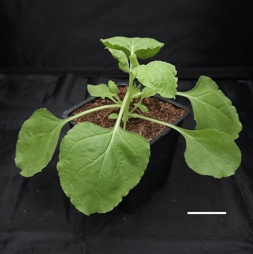


**a**

*ΔNbhexo1*


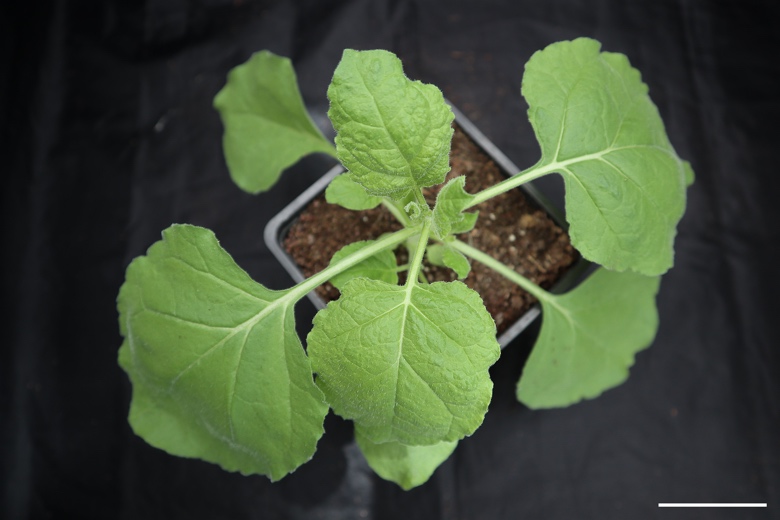

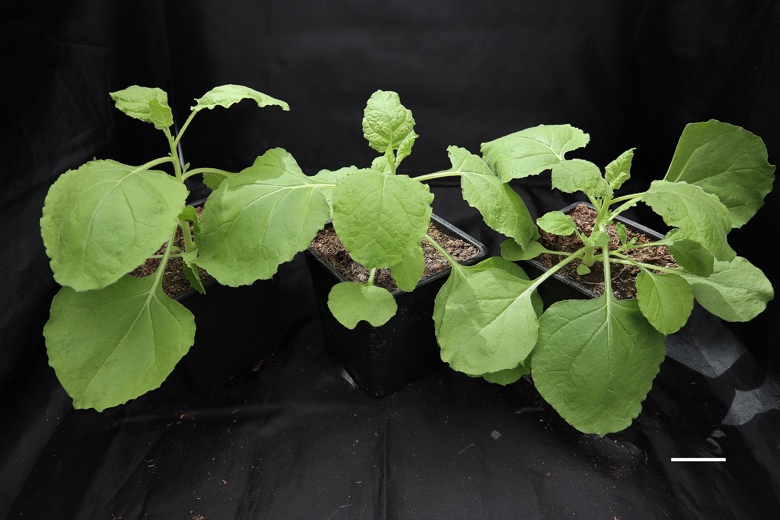

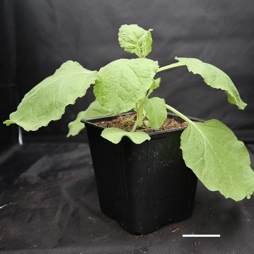

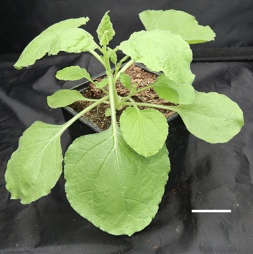


**c**


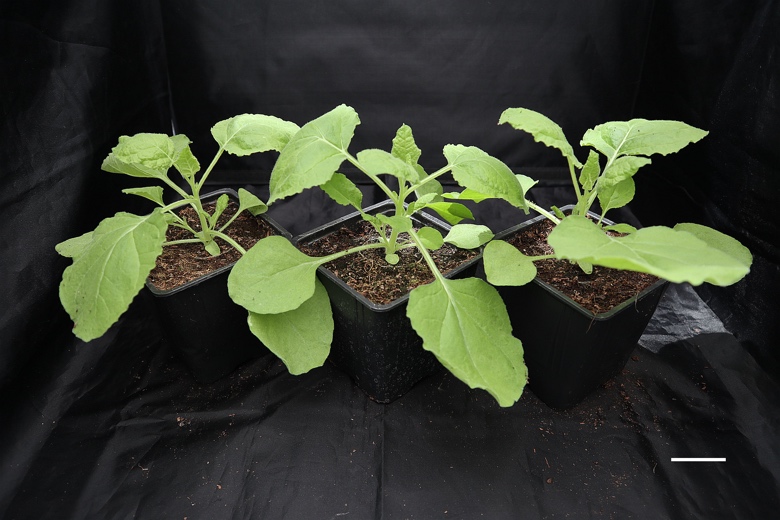


*ΔNbhexo2*


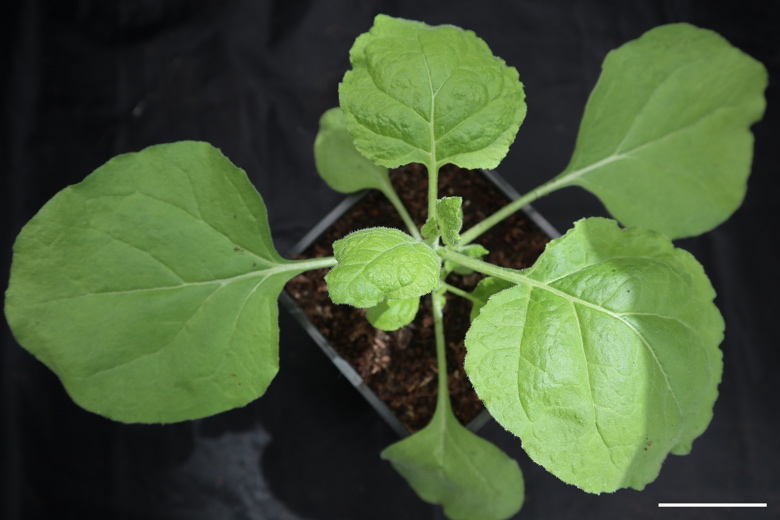

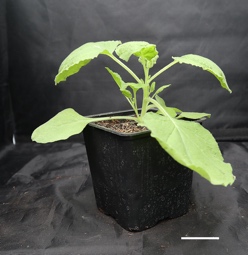

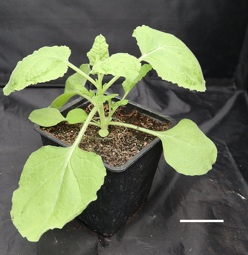


**d**


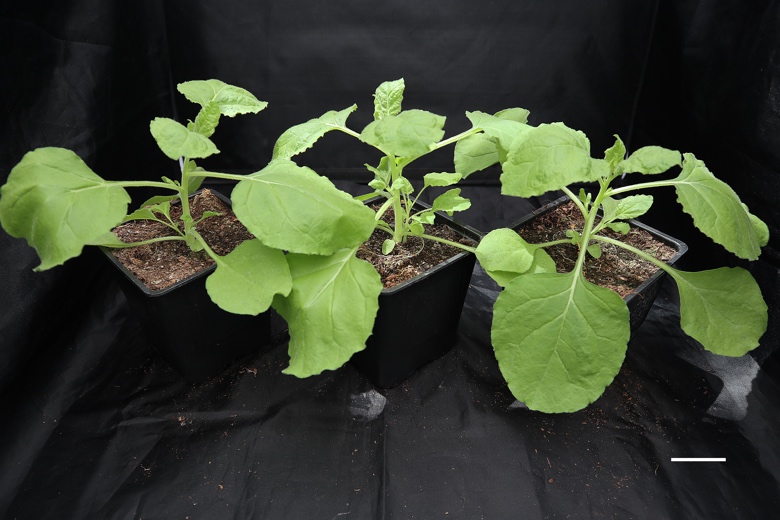


*ΔNbhexo3*


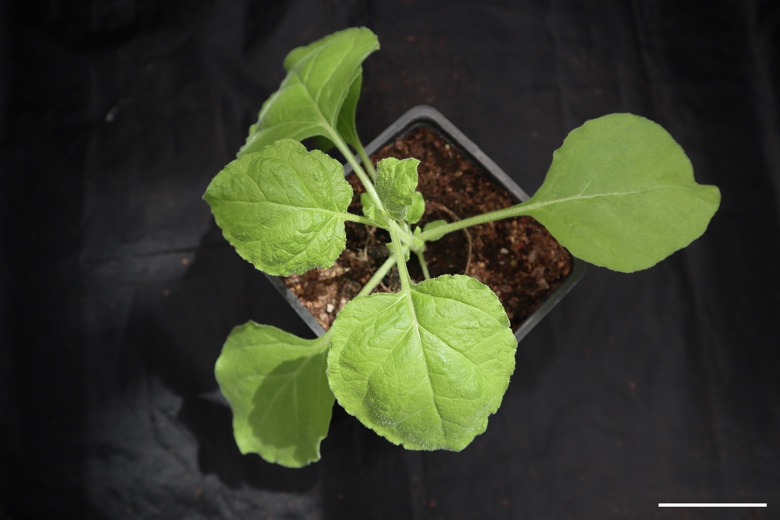

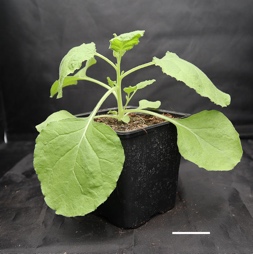

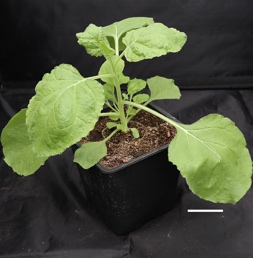


**e**

**b**


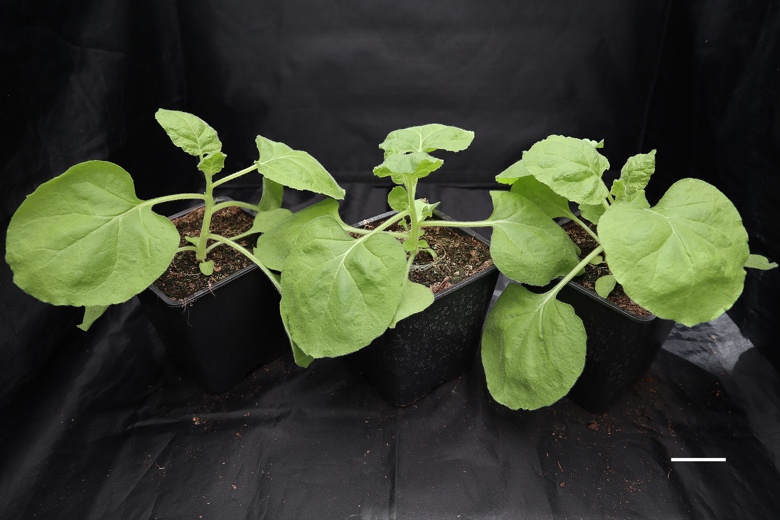


Tissue Culture Only


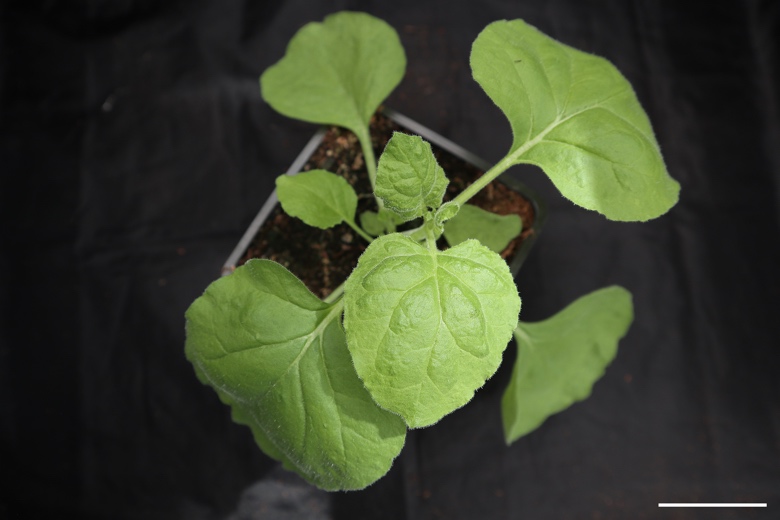

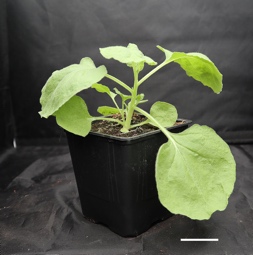


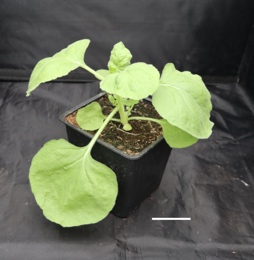

Supplement: Supplementary file 1 — Table S1 Primer sequences used in this study. Table S2 sgRNA sequences used in this study. Table S3 Synthesized sequences used in this study. [file PBI-24-239-s001.docx]
